# Efficacy and safety of traditional Chinese medicine injections combined with conventional Western medicine for post-AMI heart failure: a systematic review and Bayesian network meta-analysis of 62 randomized controlled trials

**DOI:** 10.3389/fcvm.2026.1921437

**Published:** 2026-08-19

**Authors:** Zhan-Ze Ma, Shuang Xu, Chong-Chai Li, Yao-Yao Sun, Ming-Zhu Li, Ming-Xue Zhang

**Affiliations:** 1 Department of Integrated Traditional Chinese and Western Medicine, Liaoning University of Traditional Chinese Medicine Xinglin College, Shenyang, China; 2 The First Clinical Medical College, Liaoning University of Traditional Chinese Medicine, Shenyang, China; 3 Department of Geriatrics, the Tenth Clinical Medical College of Guangzhou University of Traditional Chinese Medicine, Zhongshan Hospital of Traditional Chinese Medicine, Zhongshan, China; 4 School of Basic Medical Sciences, Liaoning University of Traditional Chinese Medicine, Shenyang, China; 5 Department of Cardiovascular Medicine Ⅱ, Affiliated Hospital of Liaoning University of Traditional Chinese Medicine, Shenyang, China

**Keywords:** acute myocardial infarction, Bayesian network meta-analysis, Chinese herbal injection, inflammatory factors, Killip classification, major adverse cardiovascular events, post-infarction heart failure, ventricular remodeling

## Abstract

**Background:**

Heart failure following acute myocardial infarction (AMI) is a prevalent and severe cardiovascular complication, which markedly elevates the risks of mortality and readmission in affected patients. Although reperfusion therapy and standardized Western medical pharmacotherapy can ameliorate clinical prognosis, a subset of patients still progress to heart failure due to ventricular remodeling and sustained neuroendocrine activation. In recent years, traditional Chinese medicine injections (TCMIs) combined with conventional western medicine (CWM) have exhibited potential merits in enhancing cardiac function, suppressing inflammatory response and delaying ventricular remodeling. Nevertheless, systematic comparisons regarding the relative efficacy and safety of various combination regimens remain insufficient. Accordingly, it is essential to systematically assess the efficacy and safety of different TCMIs plus conventional therapy for heart failure post AMI.

**Methods:**

Electronic retrievals were performed across seven databases: China National Knowledge Infrastructure, VIP Database, Wanfang Data, Chinese Biomedical Literature Database, PubMed, Web of Science and Cochrane Library, with the retrieval period spanning from January 1, 2016 to June 1, 2026. Randomized controlled trials (RCTs) investigating TCMIs combined with conventional Western regimens for heart failure after acute myocardial infarction were enrolled. Stata and R statistical software were utilized to conduct network meta-analysis.

**Results:**

A total of 62 eligible RCTs containing 5922 participants and covering 8 varieties of TCMIs were ultimately included. Network meta-analysis (NMA) demonstrated that all eight TCMIs combination regimens could significantly raise clinical response rate, modulate indicators reflecting cardiac function and myocardial injury, decrease the concentrations of inflammatory factors, accompanied by low incidence rates of major adverse cardiovascular events (MACE) and adverse reactions. The results of the surface under the cumulative ranking curve (SUCRA) indicated that Shenfu Injection plus conventional Western therapy achieved the optimal efficacy in elevating cardiac output and stroke volume, as well as reducing N-terminal pro-B-type natriuretic peptide and tumor necrosis factor-α. Xinmailong Injection ranked favorably in lowering interleukin-6 and endothelin-1. Shenmai Injection obtained the highest SUCRA rankings in boosting left ventricular ejection fraction, ameliorating left ventricular end-systolic diameter, heart rate and B-type natriuretic peptide. Yiqi Fumai Injection presented superior rankings in improving cardiac index, diastolic blood pressure, creatine kinase isoenzyme-MB and reducing the occurrence of MACE. Xuebijing Injection occupied the top SUCRA position in improving overall clinical efficacy and decreasing cardiac troponin I and high-sensitivity C-reactive protein. Shuxuening Injection ranked first in adjusting left ventricular end-diastolic diameter and systolic blood pressure, while Huangqi Injection yielded the best outcomes in improving left ventricular end-systolic volume and Left Ventricular End-Diastolic Volume. All above findings were derived from indirect comparative evidence, requiring further verification via subsequent head-to-head direct comparative trials.

**Conclusion:**

Restricted by low-certainty evidence, all eight TCMIs combined with CWM can preliminarily improve clinical efficacy, optimize parameters of cardiac function and myocardial injury, and inhibit inflammatory levels in patients with heart failure post AMI, with mild MACE and adverse events. However, the evidence network in this analysis is predominantly star-shaped without closed loops, lacking direct head-to-head comparisons among distinct TCMIs. Additionally, the included trials are limited by small sample sizes, methodological flaws and substantial clinical heterogeneity. Therefore, the current outcomes merely serve as exploratory hypotheses rather than definitive clinical conclusions. Large-sample, multicenter and high-quality RCTs are still warranted to validate these preliminary findings and generate evidence-based references for individualized precise clinical medication.

**Systematic Review Registration:**

https://www.crd.york.ac.uk/PROSPERO/view/CRD420261428750.

## 1 Introduction

Acute myocardial infarction (AMI) is one of the leading causes of cardiovascular mortality and disability worldwide (1). With the continuous advancement of percutaneous coronary intervention (PCI), thrombolytic therapy, and evidence-based pharmacological treatments, the acute mortality of patients with AMI has declined significantly (2). However, heart failure (HF) remains one of the most common and severe complications following AMI (3). Studies have shown that patients who develop HF after AMI not only experience prolonged hospitalization and increased rates of rehospitalization, but also face a substantially higher risk of long-term cardiovascular mortality (4, 5). Therefore, improving cardiac function and delaying disease progression in patients with post-AMI HF are of great importance for enhancing long-term clinical outcomes.

The development and progression of post-AMI HF involve complex pathophysiological mechanisms. Myocardial ischemic necrosis can trigger a series of pathological changes, including activation of inflammatory responses, increased oxidative stress, persistent neurohormonal overactivation, and ventricular remodeling, ultimately leading to progressive deterioration of cardiac structure and function (6, 7). Current treatment strategies are primarily based on reperfusion therapy and guideline-directed medical therapy (GDMT), including angiotensin-converting enzyme inhibitors (ACEIs), angiotensin II receptor blockers (ARBs), angiotensin receptor-neprilysin inhibitors (ARNIs), *β*-blockers, mineralocorticoid receptor antagonists, and diuretics (8, 9). Although these therapies have substantially improved prognosis, they carry risks such as bradycardia, hyperkalemia, electrolyte imbalance, and worsening heart failure, and a considerable proportion of patients continue to show progressive cardiac dysfunction and recurrent events despite standard treatment, suggesting that current strategies are insufficient to halt disease progression entirely (10–12).

As an important component of integrated traditional Chinese and Western medicine, traditional Chinese medicine injections (TCMIs) have been increasingly used in patients with post-AMI HF in recent years. Modern pharmacological studies have demonstrated that TCMIs possess the characteristics of multi-component, multi-target, and multi-pathway synergistic effects. They may exert cardioprotective effects through various mechanisms, including improving myocardial energy metabolism, promoting ischemic myocardial repair, enhancing coronary microcirculation, suppressing inflammatory responses, alleviating oxidative stress injury, and attenuating ventricular remodeling. Numerous randomized controlled trials (RCTs) have shown that the addition of TCMIs to conventional Western medicine can further improve cardiac function, reduce levels of myocardial injury biomarkers, and decrease the incidence of adverse cardiovascular events (13, 14). However, a wide variety of TCMIs are currently used in clinical practice, and the comparative efficacy and safety among different agents remain unclear.

Conventional meta-analysis is limited to direct comparisons between two interventions and is unable to simultaneously evaluate the relative advantages of multiple treatment options. In contrast, Bayesian network meta-analysis (NMA) can integrate both direct and indirect evidence, allowing the simultaneous comparison and ranking of multiple interventions and thereby providing more comprehensive evidence for clinical decision-making. Therefore, the present study conducted a Bayesian NMA to systematically evaluate the efficacy and safety of different TCMIs combined with conventional Western medicine in the treatment of post-AMI HF. In addition, the surface under the cumulative ranking curve (SUCRA) was used to rank the relative performance of different interventions, with the aim of providing evidence-based support for optimizing integrated traditional Chinese and Western medicine treatment strategies for post-AMI HF.

## 2 Materials and methods

### 2.1 Study design and registration

This study was conducted as a systematic review and Bayesian NMA to compare the efficacy and safety of different TCMIs combined with conventional western medicine (CWM) for the treatment of heart failure following acute myocardial infarction (post-AMI HF). CWM was defined as GDMT plus any additional active agents (e.g., rhBNP, dobutamine, levosimendan, dopamine, enoxaparin) administered as clinically indicated, provided that the same background regimen was applied equally to both the experimental and control groups within each original study. The study protocol was prospectively registered in the International Prospective Register of Systematic Reviews (PROSPERO) under registration number CRD420261428750. The design, conduct, analysis, and reporting of this study followed the Preferred Reporting Items for Systematic Reviews and Meta-Analyses (PRISMA) statement and its extension for network meta-analysis (PRISMA-NMA).

### 2.2 Literature search strategy

Relevant studies were identified through systematic searches of the China National Knowledge Infrastructure, Wanfang Database, VIP Database, Chinese Biomedical Literature Database, PubMed, Web of Science, and the Cochrane Library. The retrieval period covered publications from January 1, 2016 to June 1, 2026. Studies published in either Chinese or English were eligible for inclusion.

A combination of subject headings and free-text terms was applied during the literature search. The search strategy mainly included four domains: (1) disease-related terms, such as “acute myocardial infarction”, “myocardial infarction”, “acute MI”, “AMI”, “ST-segment elevation myocardial infarction”, “non-ST-segment elevation myocardial infarction”, and “acute coronary syndrome”; (2) heart failure-related terms, including “heart failure”, “cardiac insufficiency”, “congestive heart failure”, “acute heart failure”, “left ventricular failure”, and “pump failure”; (3) intervention-related terms, including “traditional Chinese medicine”, “Chinese medicine injection”, “TCM injection”, “integrated traditional Chinese and Western medicine”, and “injection”; and (4) study design-related terms, including “randomized controlled trial”, “randomized”, “controlled trial”, and “clinical trial”.

**Figure 1:**
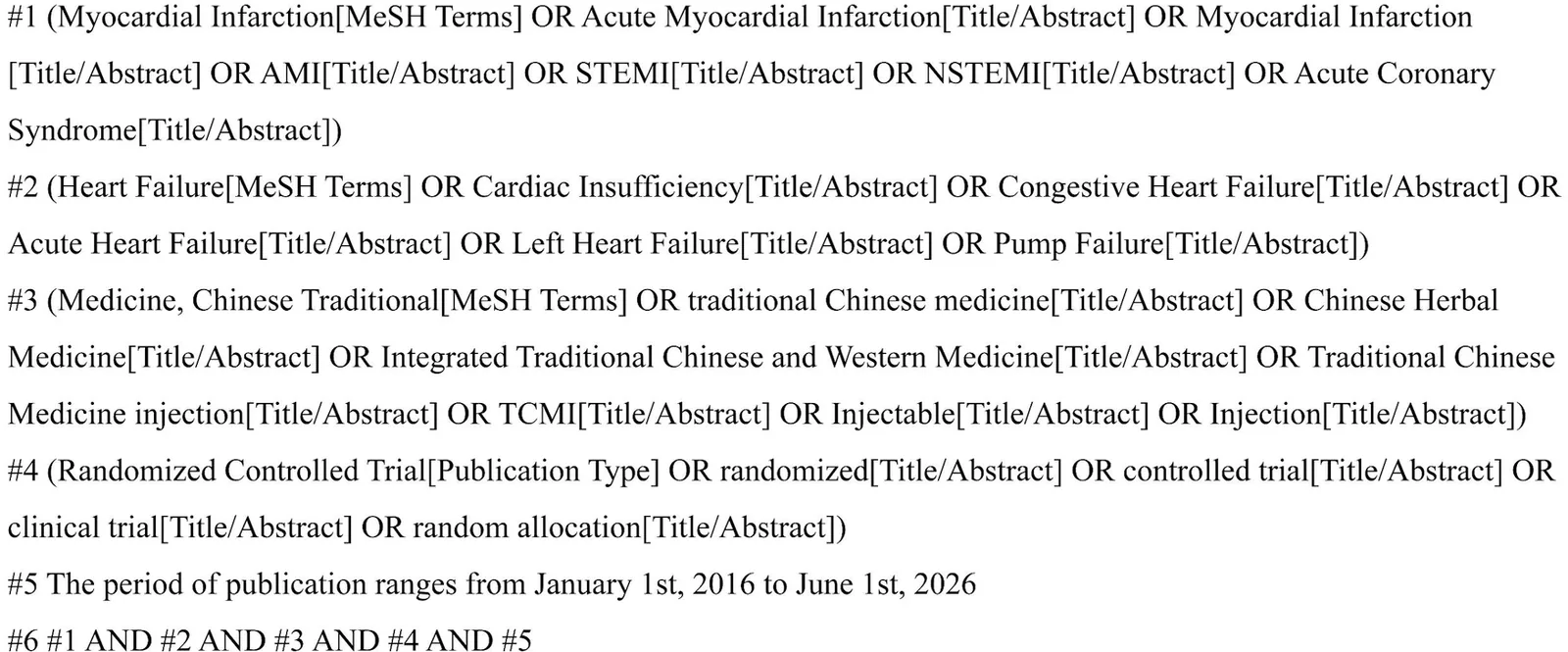
Pubmed literature search strategy.

The search strategy was adjusted appropriately according to the characteristics of individual databases. In addition, the reference lists of relevant reviews and eligible studies were manually screened. Grey literature, conference proceedings, and dissertations were also searched whenever available to minimize publication bias and reduce the possibility of missing eligible studies. Taking pubMed as an example, after literature retrieval, the results were imported into NoteExpress for literature management (Figure 1).

### 2.3 Eligibility criteria

The inclusion and exclusion criteria were established according to the PICOS framework.

#### 2.3.1 Inclusion criteria

Studies were considered eligible if they met all of the following criteria: (1) Participants: Patients diagnosed with heart failure after acute myocardial infarction according to recognized diagnostic criteria; (2) Interventions: TCM injections combined with CWM; (3) Comparators: The control group received the same CWM as that used in the experimental group; (4) Outcomes: At least one predefined outcome measure was reported; (5) Study design: RCTs; (6) Language: Studies published in Chinese or English.

#### 2.3.2 Exclusion criteria

Studies meeting any of the following criteria were excluded: (1) Duplicate publications; (2) Animal experiments, basic laboratory studies, case reports, reviews, systematic reviews, or meta-analyses; (3) Non-randomized studies; (4) Studies in which additional traditional Chinese medicine therapies, such as herbal decoctions, acupuncture, or moxibustion, were simultaneously administered in the experimental group, making it impossible to independently evaluate the efficacy of TCMIs; (5) Studies with unavailable full texts, incomplete data, or data that could not be extracted; (6) Studies with obvious methodological defects or unclear outcome reporting.

#### 2.3.3 Definition of intervention nodes

To ensure consistency in network construction, intervention nodes were defined according to the type of TCMIs. Preparations containing the same TCMIs but produced by different manufacturers or administered at different dosages were classified as the same intervention node. Studies involving the simultaneous use of two or more TCMIs were excluded from the network meta-analysis. All interventions were administered on the basis of CWM in order to minimize the influence of differences in background therapy. Although Shenmai Injection and Shengmai Injection have similar English transliterations, they are two distinct traditional Chinese medicine injections with different pharmaceutical compositions and formulations. Therefore, they were classified as separate network nodes in the present analysis.

### 2.4 Literature screening and data extraction

All retrieved records were imported into NoteExpress for management and duplicate removal. Two investigators independently screened the studies by title, abstract, and full text according to predefined eligibility criteria. Potentially eligible or unclear records were retrieved for full-text assessment. Disagreements were resolved through discussion, with arbitration by a third investigator when necessary.

A standardized data extraction form was developed prior to data collection. Extracted information included first author, publication year, sample size, age, sex, Killip classification, intervention details, treatment duration, outcome indicators, adverse events, and methodological quality domains (randomization, allocation concealment, blinding, and completeness of outcome data). All extracted data were cross-checked by the two investigators to ensure accuracy.

To prevent double-counting of the same patient cohort, we performed additional manual verification beyond automated duplicate removal. For studies with similar sample sizes, outcomes, or authorship teams, we compared institutional affiliations, enrollment periods, and baseline characteristics. Only studies conducted at distinct centers or during non-overlapping recruitment periods were considered independent. Studies from the same institution with overlapping enrollment periods were carefully examined, and the less informative or earlier version was excluded. This process ensured that all included trials represented independent patient populations.

### 2.5 Outcome measures

To comprehensively evaluate the efficacy and safety of different TCMIs combined with CWM in patients with post-AMI HF, outcome measures were classified according to their clinical significance.

#### 2.5.1 Primary outcomes

Primary outcomes included: (1) Clinical efficacy; (2) Left ventricular ejection fraction (LVEF); (3) Major adverse cardiovascular events (MACE). MACE outcomes were extracted according to the definitions reported in each original trial. The components and follow-up durations of MACE varied among studies.

#### 2.5.2 Secondary outcomes

Secondary outcomes included: Cardiac structure and function indicators: left ventricular end-diastolic diameter (LVEDD), left ventricular end-systolic diameter (LVESD), left ventricular end-diastolic volume (LVEDV), left ventricular end-systolic volume (LVESV), cardiac output (CO), cardiac index (CI), and stroke volume (SV); Hemodynamic indicators: heart rate (HR), systolic blood pressure (SBP), and diastolic blood pressure (DBP); Myocardial injury biomarkers: creatine kinase-MB (CK-MB) and cardiac troponin I (cTnI); Heart failure biomarkers: B-type natriuretic peptide (BNP) and N-terminal pro-B-type natriuretic peptide (NT-proBNP); Inflammatory and endothelial function indicators: high-sensitivity C-reactive protein (hs-CRP), tumor necrosis factor-α (TNF-α), interleukin-6 (IL-6), and endothelin-1 (ET-1).

### 2.6 Risk of bias assessment

The methodological quality of the included studies was independently evaluated by two investigators using the Cochrane Risk of Bias Tool (RoB 2.0). The assessment domains included random sequence generation, allocation concealment, blinding of participants and personnel, blinding of outcome assessment, completeness of outcome data, selective reporting, and other potential sources of bias. Each domain was judged as having a low, high, or unclear risk of bias according to the recommendations of the Cochrane Handbook. Any discrepancies between investigators were resolved through discussion or consultation with a third reviewer.

### 2.7 Statistical analysis

Review Manager 5.4, Stata 18.0, R software (Version 4.5.2), and JAGS software (Version 4.3.2) were used for statistical analyses. Odds ratios (ORs) with 95% credible intervals (CrIs) were calculated for dichotomous outcomes. For continuous outcomes, we used change-from-baseline values as the primary data source. Studies that reported only post-intervention absolute values without providing baseline measurements or sufficient data to calculate the change-from-baseline standard deviation were excluded from the corresponding NMA. For studies reporting both baseline and endpoint means and standard deviations without the change score standard deviation, we imputed the latter using the formula SD(change) = √[SD(baseline)^2^ + SD(final)^2^−(2 × R × SD(baseline) × SD(final))], with a correlation coefficient of R = 0.5 assumed for imputation. Given that the measurement units for each outcome were consistently reported across studies, we used mean difference as the effect measure. Logarithmic transformation was not applied to any biomarker outcome, as the between-study variance was adequately captured by the random-effects model.

Pairwise meta-analyses were initially performed for outcomes with direct comparison data. Subsequently, a Bayesian random-effects network meta-analysis was conducted using Markov chain Monte Carlo (MCMC) methods. Three Markov chains with different initial values were simultaneously run. The burn-in period was set at 20,000 iterations, followed by 50,000 sampling iterations. Model convergence was assessed using the potential scale reduction factor (PSRF). A PSRF value approaching 1.00 was considered indicative of satisfactory convergence.

Network plots were generated to illustrate the evidence structure among different interventions. In these plots, node size was proportional to the cumulative sample size, whereas edge thickness reflected the number of direct comparisons. The node-splitting method was employed to evaluate the consistency between direct and indirect evidence. A consistency model was adopted when no statistically significant inconsistency was detected (*P* > 0.05). Otherwise, potential sources of inconsistency were further explored. The surface under the cumulative ranking curve (SUCRA) was calculated to rank the efficacy of different interventions. Higher SUCRA values indicated a greater probability that an intervention would be among the most effective treatment options.

Statistical heterogeneity was assessed using the I^2^ statistic. An I^2^ value greater than 50% was considered indicative of substantial heterogeneity. Potential sources of heterogeneity were explored based on clinical characteristics, treatment regimens, treatment duration, and outcome assessment timing. When at least 10 studies were available for a given outcome, comparison-adjusted funnel plots and Egger's regression test were used to assess publication bias. A *P* value < 0.05 was considered suggestive of potential publication bias.

### 2.8 Certainty of evidence assessment

The certainty of evidence for major outcomes was assessed using the Confidence in Network Meta-Analysis (CINeMA) framework. Six domains were evaluated, including within-study bias, reporting bias, indirectness, imprecision, heterogeneity, and incoherence. Based on the overall assessment across these domains, the certainty of evidence was categorized as high, moderate, low, or very low. This evaluation was used to determine the robustness and reliability of the findings derived from the network meta-analysis.

## 3 Results

### 3.1 Literature search results

**Figure 2:**
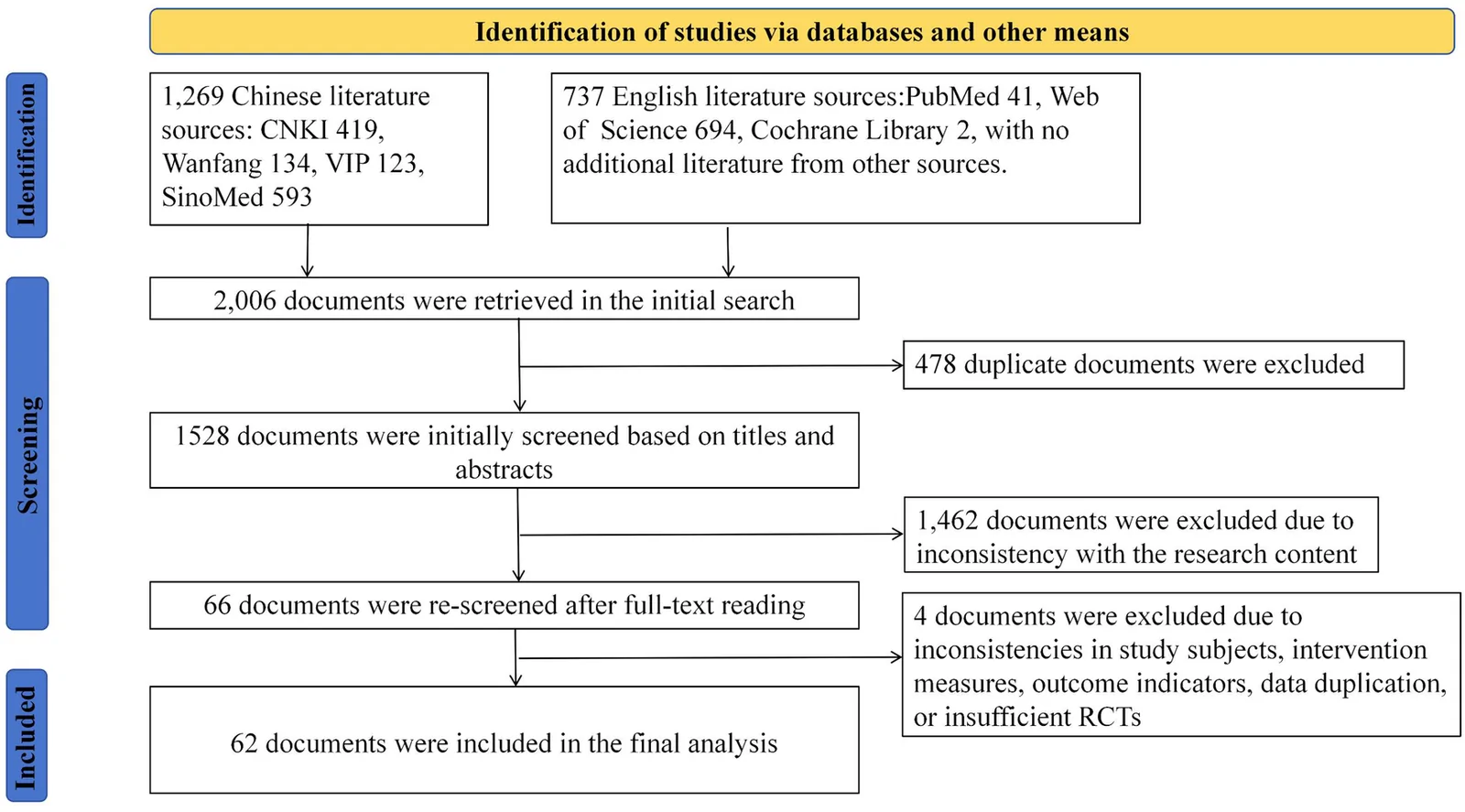
Literature screening process.

A total of 2,006 studies were retrieved from the above databases according to the search strategy, including 1,269 Chinese-language articles and 737 English-language articles. Based on the inclusion and exclusion criteria, 1,944 studies were excluded, and a total of 62 studies were finally included, all of which were Chinese-language publications (Figure 2).

### 3.2 Basic characteristics of included studies

A total of 62 studies (16, 77) were included, all of which were two-arm trials, involving 5,922 patients in total, with 2,969 patients in the experimental group and 2,953 patients in the control group. A total of 8 types of TCMIs were involved, including Shenfu Injection (SFI) in 19 studies (16–34); Xinmailong Injection (XMLI) in 13 studies (35–47); Shenmai Injection (SMI(a)) in 8 studies (48–55); Yiqi Fumai Injection (YQFMI) in 7 studies (56–62); Xuebijing Injection (XBJI) in 6 studies (63–68); Shengmai Injection (SMI(b)) in 4 studies (69–72); Shuxuening Injection (SXNI) in 3 studies (73–75) and Huangqi Injection (HQI) in 2 studies (76, 77) (Table 1, Supplementary Table S1).

**Table 1:** Basic characteristics of included literature.

First author and year	Samplesize (n)		Male and female case numbers(n)		Age(years)		Killip classification		Treatment group intervention	Control group intervention	Treatment course	Outcome measures
	T	C	T	C	T	C	T	C				
YOU S et al., 2019 (16)	38	38	21/17	20/18	54.67 ± 9.68	52.35 ± 10.27	II/III	II/III	SFI + CG	rhBNP + CWMCWM	7 days	①⑮⑳
LI X et al., 2016 (17)	23	23	17/6	14/9	68.6 ± 2.6	68.7 ± 2.6	III/IV	III/IV	SFI + CG	CWMCWM	14 days	①②⑦⑧⑩⑭⑰⑱
WANG SN, 2021 (18)	33	32	21/12	22/10	73 ± 12.8	72 ± 13.6	II/III	II/III	SFI + CG	CWMCWM	10 days	①②⑦⑭
ZHANG ZX et al., 2018 (19)	38	38	21/17	20/18	63.32 ± 1.78	63.91 ± 5.86	I-III	I-III	SFI + CG	CWMCWM	28 days	①②⑦⑧⑩⑰⑱
WANG CL et al., 2017 (20)	64	64	36/28	38/26	59.7 ± 14.3	58.2 ± 13.6	II-IV	II-IV	SFI + CG	DA + CWMCWM	7 days	①⑩⑪⑫⑭
WU W et al., 2017 (21)	64	64	36/28	38/26	59.7 ± 14.3	58.2 ± 13.6	II-IV	II-IV	SFI + CG	DA + CWM	7 days	①⑩⑪⑫⑭
FENG JP et al., 2019 (22)	174	160	107/67	90/70	60.79 ± 9.73	61.43 ± 7.22	III/IV	III/IV	SFI + CG	Enoxaparin+ CWM	10 days	②③⑬⑮⑳㉑
TAN CL et al., 2024 (23)	40	40	28/12	22/18	62.80 ± 12.54	66.22 ± 11.15	-	-	SFI + CG	CWM	7 days	②
MA XG et al., 2019 (24)	55	55	32/23	31/24	59.14 ± 4.21	59.12 ± 4.22	I-III	I-III	SFI + CG	CWM	14 days	①②㉑
LI R et al., 2017 (25)	31	31	18/13	16/15	66.38 ± 10.69	67.41 ± 11.98	III/IV	III/IV	SFI + CG	CWM	14 days	①②⑩⑭㉑
ZHANG YH et al., 2019 (26)	33	32	20/13	21/11	20–85	18–84	II-IV	II-IV	SFI + CG	CWM	10 days	⑭⑰㉒
YANG XJ et al., 2019 (27)	50	50	27/23	29/21	56.19 ± 2.08	57.29 ± 3.28	II-IV	II-IV	SFI + CG	Dobutamine+ CWM	3 days	①⑦⑧⑨
ZHAO J et al., 2016 (28)	31	31	32/30		68 ± 5		II/III	II/III	SFI + CG	rnBNP + GDMT	7 days	②③⑩⑪⑫⑮⑰⑳㉑㉒
ZHANG DC et al., 2018 (29)	35	35	16/19	17/18	66.3 ± 8.7	65.7 ± 8.4	II-IV	II-IV	SFI + CG	CWM	7 days	②⑤⑩⑮⑰⑳㉑
WANG HY et al., 2018 (30)	30	31	-	-	-	-	II/III	II/III	SFI + CG	rhBNP + CWM	7 days	②③⑩⑪⑫⑮⑰⑳㉑㉒
SUN YX, 2016 (31)	31	31	16/15	20/11	65.3 ± 5.1	67.1 ± 5.3	-	-	SFI + CG	rhBNP + CWM	7 days	⑩⑪⑫
WANG YT, 2018 (32)	58	58	35/23	36/22	60.8 ± 2.5	64.8 ± 2.5	-	-	SFI + CG	rhBNP + CWM	7 days	⑩⑪⑫
ZHOU Q, 2023 (33)	54	54	31/23	29/25	52.58 ± 4.33	53.02 ± 4.46	-	-	SFI + CG	rhBNP + CWM	10 days	③
WANG DQ, 2019 (34)	45	45	27/18	26/19	55.2 ± 3.6	55.1 ± 3.7	II/III	II/III	SFI + CG	rhBNP + CWM	3 days	③④⑤⑥⑬⑮⑯⑰⑱⑲
LI W, 2017 (35)	50	50	19/31	22/28	54.92 ± 5.83	55.27 ± 6.75	-	-	XMLI + CG	CWM	14 days	①⑭⑲
WEI JJ, 2023 (36)	42	42	25/17	27/15	44.42 ± 3.23	43.57 ± 3.03	-	-	XMLI + CG	rhBNP + CWM	10 days	②③④⑮⑲
YAN RY et al., 2019 (37)	61	61	37/24	39/22	87.12 ± 6.98	86.35 ± 7.67	II-IV	II-IV	XMLI + CG	CWM	10 days	②⑭⑯⑰⑳
LIU DS et al., 2020 (38)	46	46	30/16	28/18	60.4 ± 11.3	61.2 ± 10.9	-	-	XMLI + CG	CWM	7 days	②⑮⑯㉒
LIU YL et al., 2019 (39)	76	76	48/28	40/36	61.35 ± 6.72	63.85 ± 6.12	-	-	XMLI + CG	CWM	10–20 days	②③④⑦⑨⑩⑭⑯
YUAN XY, 2019 (40)	43	43	27/16	29/14	67.85 ± 8.97	68.02 ± 9.35	II-IV	II-IV	XMLI + CG	CWM	7 days	②⑭⑱⑲
XU H et al., 2020 (41)	35	35	22/13	20/15	70.23 ± 11.94	73.74 ± 11.9	II-IV	II-IV	XMLI + CG	CWM	5 days	①
LI TT et al., 2016 (42)	23	23	28/18		56.87 ± 5.34		III/IV	III/IV	XMLI + CG	GDMT	14 days	①②⑭⑯⑰⑲
SONG FF et al., 2022 (43)	60	60	38/22	35/25	69.1 ± 4.0	68.3 ± 4.3	-	-	XMLI + CG	CWM	7 days	②③④⑨⑮⑰⑱⑲
WANG WG et al., 2024 (44)	53	53	34/19	32/21	65.41 ± 5.14	65.41 ± 5.14	II-IV	II-IV	XMLI + CG	Bisoprolol Fumarate + CWM	14 days	①⑬⑮⑰⑲㉒
WANG Q, 2019 (45)	49	49	23/26	22/27	72.09 ± 8.26	71.83 ± 8.42	-	-	XMLI + CG	Levosimendan + CWM	14 days	①②⑮
ZHANG XH, 2016 (46)	35	35	22/13	20/15	≥80	≥80	II-IV	II-IV	XMLI + CG	CWM	10 days	②⑮⑯
LI JF et al., 2016 (47)	50	50	37/13	34/16	85 ± 4.6	84 ± 5.1	III/IV	III/IV	XMLI + CG	CWM	14 days	②⑭
XU XJ et al., 2016 (48)	38	38	22/16	21/17	62.7 ± 8.9	62.1 ± 8.6	II-IV	II-IV	SMI + CG(a)	Dobutamine+ CWM	10 days	①②⑩
ZHANG J et al., 2025a (49)	44	44	25/19	26/18	56.87 ± 6.35	57.89 ± 6.01	-	-	SMI + CG(a)	Creatine Phosphate Sodium + CWM	14 days	①②③④⑬⑮⑯㉑
LYU YJ et al., 2021 (50)	47	46	27/20	25/21	65.7 ± 11.9	66.0 ± 12.6	II/III	II/III	SMI + CG(a)	CWM	14 days	②⑮⑯⑰㉒
YANG JW et al., 2017 (51)	38	38	25/13	21/17	35.4 ± 6.7	36.8 ± 5.4	II/III	II/III	SMI + CG(a)	CWM	14 days	①②③⑭
ZHENG Y, 2016 (52)	34	34	19/15	21/13	64.3 ± 4.6	66.5 ± 4.7	III/IV	III/IV	SMI + CG(a)	CWM	28 days	㉑
CUI YS, 2017 (53)	40	40	24/16	23/17	66.42 ± 4.17	66.35 ± 4.62	-	-	SMI + CG(a)	CWM	28 days	②③⑭㉑
ZHAO Y et al., 2016 (54)	105	105	59/46	61/44	61.8 ± 9.5	60.2 ± 10.6	II-IV	II-IV	SMI + CG(a)	CWM	14 days	①②③⑭㉑
LI XY, 2019 (55)	40	40	23/17	22/18	66.09 ± 11.26	65.83 ± 11.42	-	-	SMI + CG(a)	Milrinone+ CWM	5 days	①㉒
QIU BB et al., 2024 (56)	48	48	25/23	28/20	57.2 ± 6.94	56.89 ± 6.8	II-IV	II-IV	SMI + CG(a)	rhBNP + CWM	7 days	②⑮⑰
ZHANG YJ et al., 2016 (57)	124	124	71/53	68/56	60.27 ± 5.81	59.24 ± 6.15	-	-	SMI + CG(a)	rhBNP + CWM	-	①②⑮⑯
LI X et al., 2021 (58)	45	45	25/20	23/22	58.79 ± 6.62	59.08 ± 6.77	II-IV	II-IV	YQFMI + CG	CWM	7 days	②④③⑭㉒
QIU LQ et al., 2024 (59)	50	50	25/25	24/26	54.27 ± 3.08	55.13 ± 3.39	II-IV	II-IV	YQFMI + CG	rhBNP + CWM	14 days	①②⑥⑤
GONG KW et al., 2023 (60)	35	35	18/17	16/19	62.83 ± 5.58	63.57 ± 5.73	-	-	YQFMI + CG	CWM	10 days	①②⑦⑧⑨⑬⑮⑯⑱㉒
LI XF et al., 2016 (61)	39	39	22/17	21/18	67 ± 8	69 ± 7	-	-	YQFMI + CG	Dobutamine+ CWM	7 days	①②⑨⑮㉑㉒
CHEN J et al., 2018 (62)	43	43	-	-	-	-	II-IV	II-IV	YQFMI + CG	CWM	7–10 days	⑩⑪⑫⑮
YAO JH et al., 2024 (63)	40	40	23/17	22/18	59.35 ± 4.36	58.8 ± 6.16	-	-	XBJI + CG	Sacubitril/Valsartan + CWM	14 days	②⑦⑳
LI YQ et al., 2023 (64)	43	43	23/20	24/19	58.39 ± 6.34	59.12 ± 6.2	III/IV	III/IV	XBJI + CG	CWM	14 days	①②⑦⑰⑱⑲⑳
LU X et al., 2024 (65)	38	38	23/15	21/17	60.13 ± 6.89	58.74 ± 7.54	II-IV	II-IV	XBJI + CG	Sacubitril/Valsartan + CWM	12 days	②⑮㉒
WANG QF, 2024 (66)	35	35	15/20	16/19	58.46 ± 6.21	58.37 ± 6.34	-	-	XBJI + CG	Sacubitril/Valsartan + CWM	-	②⑦⑳㉒
ZHANG J et al., 2025b (67)	47	47	25/22	27/20	49.17 ± 6.46	48.37 ± 6.2	-	-	XBJI + CG	Levosimendan + CWM	30 days	①②③⑬⑮⑯㉒
LI MJ, 2025 (68)	40	40	24/16	22/18	57.33 ± 5.51	57.28 ± 5.46	-	-	XBJI + CG	CWM	14 days	①②③④⑦⑮⑯㉒
WANG LM et al., 2019 (69)	53	53	29/24	31/22	59.39 ± 10.25	59.43 ± 10.31	II-IV	II-IV	SMI + CG(b)	rhBNP + CWM	5 days	①②③⑦⑨⑬⑮⑰⑲
LU DX et al., 2022 (70)	45	45	21/24	23/22	57.74 ± 7.28	58.67 ± 7.34	II/III	II/III	SMI + CG(b)	Dobutamine+ CWM	15 days	①②⑦⑧⑨⑰⑲㉒
YANG QJ, 2016 (70)	40	40	23/17	25/15	57.3	55.9	-	-	SMI + CG(b)	CWM	10 days	①②③⑭
LUAN GX et al., 2022 (72)	55	55	33/22	35/20	68.60 ± 3.02	68.75 ± 3.12	-	-	SMI + CG(b)	CWM	10 days	①②③⑨
WANG HX, 2022 (73)	35	35	23/12	25/10	61.04 ± 2.21	61.21 ± 2.34	-	-	SXNI + CG	Isosorbide mononitrate+ CWM	14 days	①②
LIU HL et al., 2021 (74)	40	40	26/14	22/18	59.32 ± 5.47	59.41 ± 5.54	-	-	SXNI + CG	Isosorbide mononitrate+ CWM	28 days	②⑩⑪⑫
QIN LY et al., 2019 (75)	41	41	21/20	22/19	61.32 ± 3.5	60.25 ± 3.26	-	-	SXNI + CG	Isosorbide mononitrate+ CWM	21 days	①②③⑩⑮⑳
XIAN W, 2019 (76)	48	48	26/22	27/21	65.27 ± 7.1	64.58 ± 7.32	II/III	II/III	HQI + CG	rhBNP + CWM	14 days	①②⑤⑥⑮⑰㉒
FU J, 2021 (77)	49	49	25/24	22/27	69.12 ± 4.03	68.52 ± 3.46	-	-	HQI + CG	rhBNP + CWM	14 days	②⑤⑥⑮⑰

①clinical efficacy; ②LVEF; ③LVEDD; ④LVESD; ⑤LVESV; ⑥ LVEDV; ⑦CO; ⑧CI; ⑨SV; ⑩HR; ⑪SBP; ⑫DBP; ⑬CK-MB; ⑭BNP; ⑮NT-proBNP; ⑯cTnI; ⑰hs-CRP; ⑱TNF-α; ⑲IL-6; ⑳ET-1㉑MACE; ㉒Adverse reactions; “-” indicates not mentioned.

rhBNP, Recombinant human B-type Natriuretic Peptide; DA, Dopamine.

### 3.3 Risk of bias assessment for included studies

**Figure 3:**
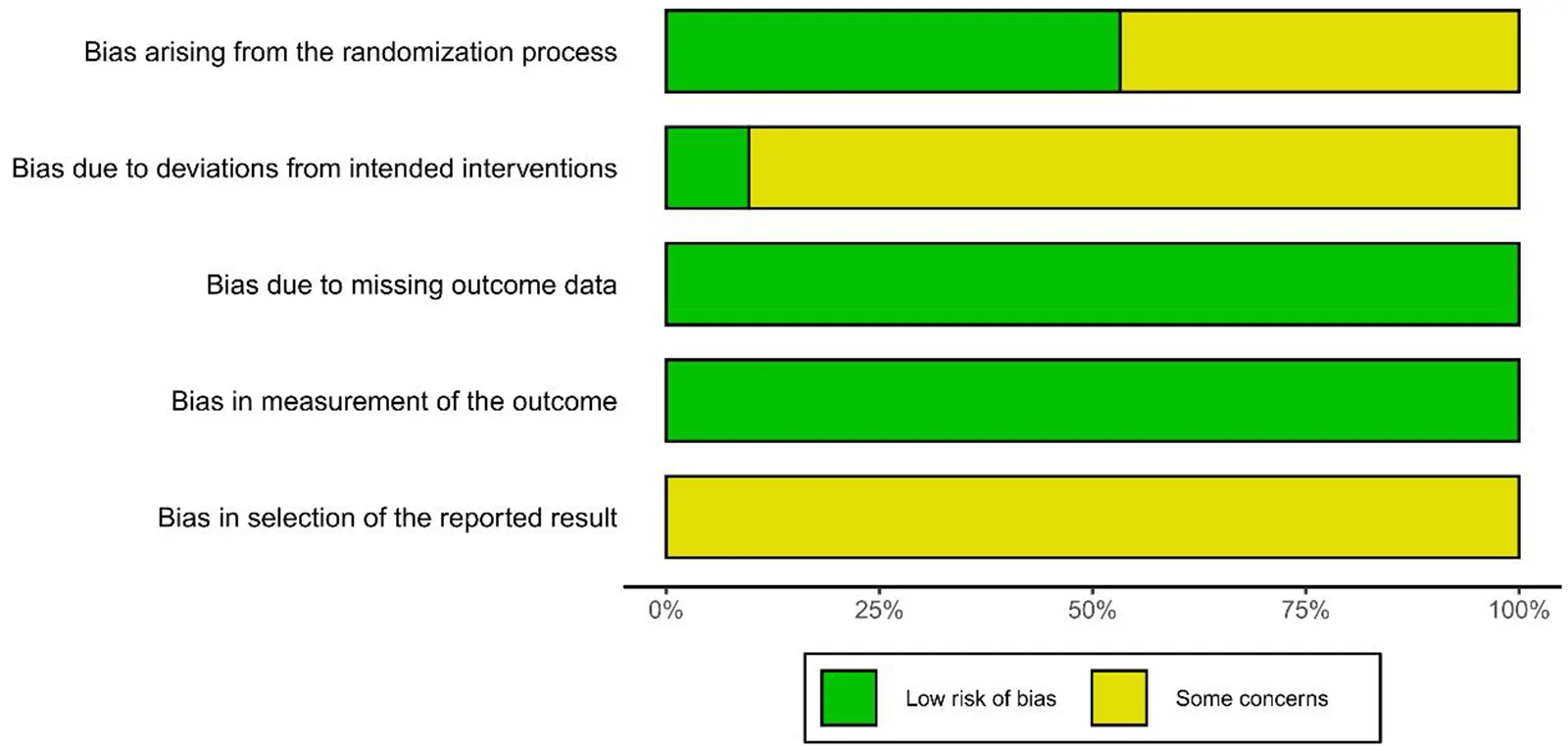
Risk of bias of the included studies.

A total of 62 RCTs were included in this study. Among them, 32 studies (16, 18, 20, 21, 23, 24, 26, 29, 32, 36, 38–40, 44, 45, 49, 50, 52, 55, 56, 59, 60, 64, 67–73, 75, 76) used the random number table method; one study adopted the coin-flipping method (17) and another used the envelope method (43), and these randomization approaches were all judged as low risk of bias. The remaining studies only mentioned random grouping without specifying detailed randomization schemes, and were rated as unclear risk of bias. None of the included studies described the implementation details of allocation concealment, so this domain was uniformly assessed as unclear risk of bias. Four studies (37, 41, 42, 46) reported single-blinding design and one study (77) mentioned double-blinding design, which were categorized as low risk of bias for this domain; the remaining studies did not report blinding implementation information and were rated as unclear risk of bias. All studies were assessed as low risk of bias in terms of incomplete outcome data. No selective reporting was detected in any included studies, resulting in a low-risk rating for this domain. No other potential sources of bias existed in all literatures, and the other bias domain was rated as low risk of bias (Figure 3, Supplementary Figure S1).

### 3.4 Network plot of evidence

**Figure 4:**
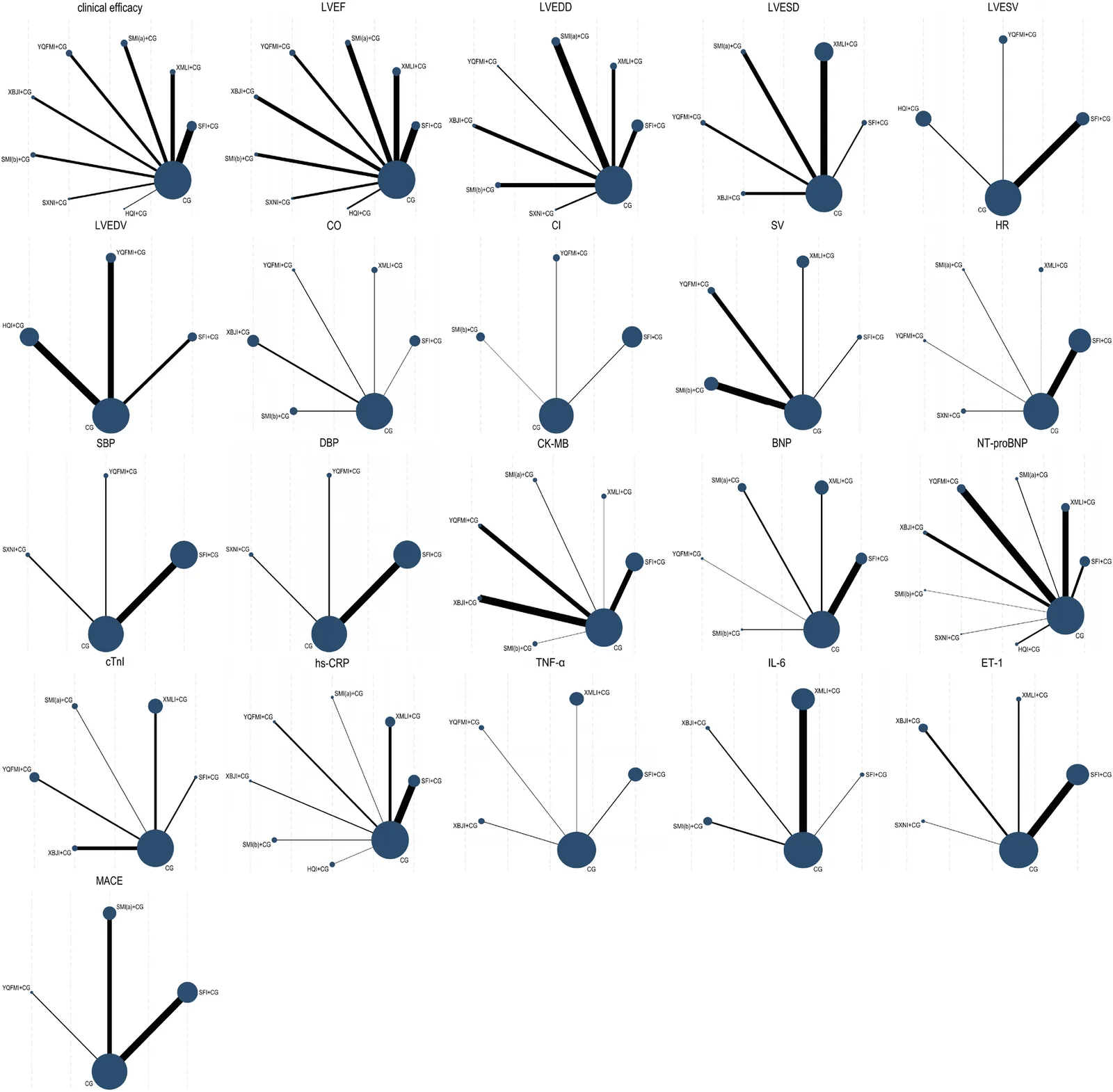
Network evidence plots for all outcome indicators.

Eight TCMIs for post-AMI HF were included in this analysis, and network evidence plots were constructed based on all assessed endpoints (Figure 4). Each node in these plots represents a separate intervention, with its size proportional to the total sample size; larger nodes indicate a greater number of participants enrolled in that treatment. Lines connecting different nodes represent head-to-head comparisons between distinct interventions. The thickness of each line is related to the number of eligible controlled trials for the pairwise comparison, and thicker lines indicate more comparative studies between the two interventions.

### 3.5 Results of network meta-analysis

#### 3.5.1 Consistency assessment via node-splitting method

Figure 4 presents the evidence network plots generated for all predefined efficacy and safety endpoints. In the current analysis enrolling eight varieties of TCMIs combined with CWM for post-AMI HF, no head-to-head RCTs were retrieved to supply direct comparative evidence between any two distinct TCMI combination regimens. Each injection intervention only formed pairwise connections with the CWM-alone control arm, eventually forming a typical star-shaped evidence network lacking closed triangular loops. Such network architecture cannot support the node-splitting approach to quantitatively test inconsistency between direct and indirect evidence. Accordingly, formal inconsistency statistics were unable to be calculated. All inter-group comparisons across different TCMIs merely relied on indirect evidence anchored to the unified CWM reference group, whereas no direct trial data could be used to externally verify the credibility of these indirect comparative outputs. Given the above structural limitation of the evidence network, a consistent Bayesian random-effects model was adopted to integrate the effect sizes of multiple interventions. Nevertheless, potential incoherent bias interfering with pooled effect estimates and SUCRA hierarchical results cannot be fully ruled out, so the ranking results derived purely from indirect comparisons should be interpreted prudently when guiding clinical medication decisions.

#### 3.5.2 Convergence diagnosis of Bayesian network models

All Bayesian network meta-analyses were executed in R software (Version 4.5.2) with the gemtc package under the MCMC framework. Three independent Markov chains with disparate starting values were simultaneously simulated, including 1,000 adaptive tuning iterations and an additional 1,000 burn-in iterations to eliminate unstable early sampling data. The overall sampling iteration was configured to 10,000 with a thinning interval of 1, yielding 9,000 valid posterior samples after discarding burn-in sequences. The PSRF served as the primary metric to judge model convergence. For all measured endpoints, including overall clinical response rate, cardiac structural and functional parameters, hemodynamic indicators, myocardial injury markers, heart failure biomarkers, as well as inflammatory and endothelial-related indices, the calculated PSRF values fluctuated between 1.00 and 1.05, all below the standard threshold of 1.1. Moreover, the trace plots of three Markov chains exhibited substantial overlapping trends without abrupt oscillations, which demonstrated satisfactory convergence performance and stable posterior parameter estimation. Collectively, the dataset included in this study met the statistical requirements for subsequent integrated effect calculation and SUCRA probability ranking analysis.

#### 3.5.3 Clinical efficacy

**Figure 5:**
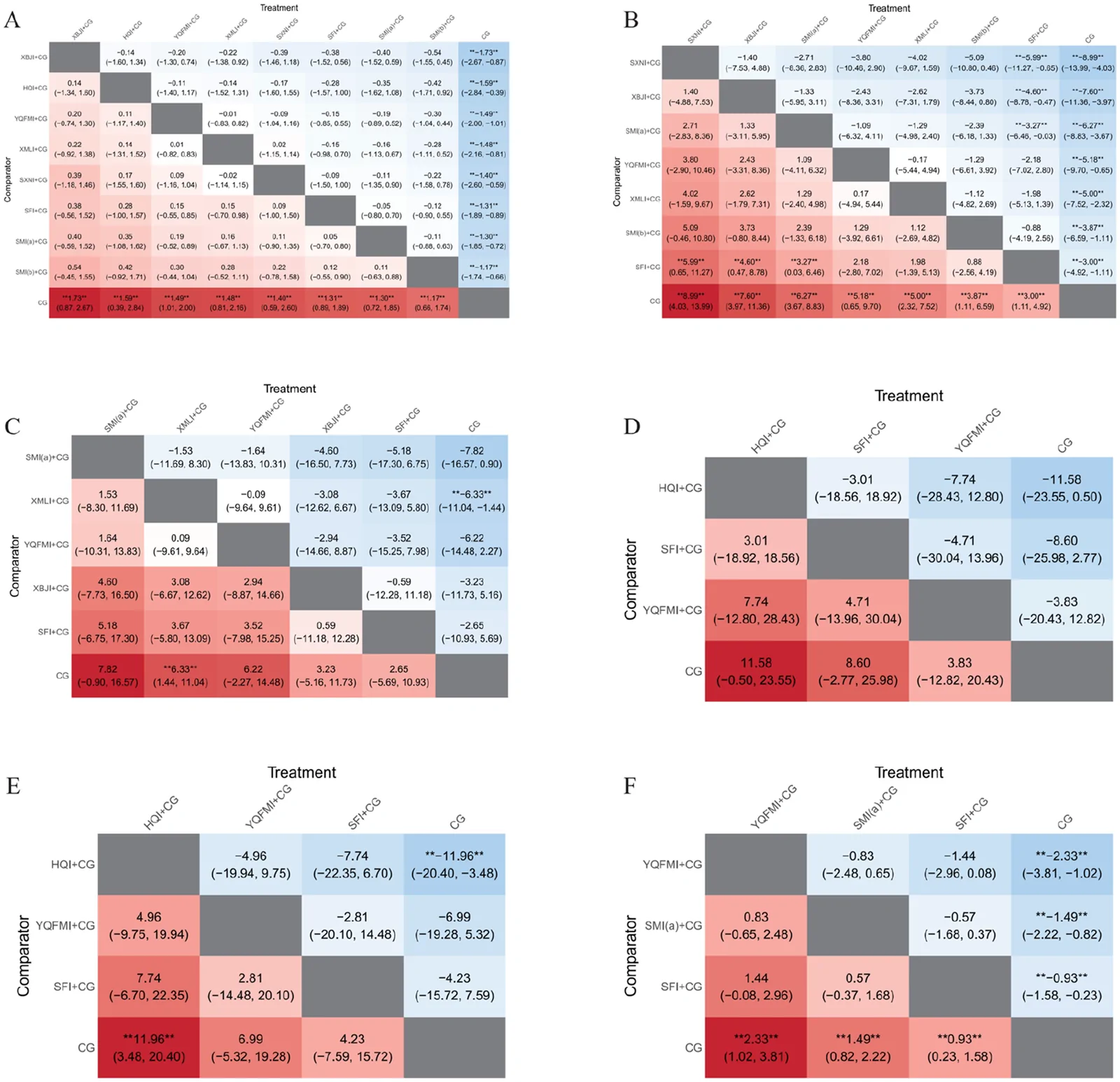
Heatmaps of clinical efficiency, cardiac structure and function indicators and MACE. **(A)** Clinical efficiency; **(B)** LVEDD; **(C)** LVESD; **(D)** LVESV; **(E)** LVEDV; **(F)** MACE. SMI(a): Shenmai Injection; SMI(b): Shengmai Injection.** indicates a statistically significant difference between the two interventions.

A total of 33 studies (16–21, 24, 25, 27, 35, 41, 42, 44, 45, 48, 49, 51, 54, 55, 57, 59–61, 64, 67–73, 75, 76) involving 3,131 subjects and 8 types of TCMIs were included in this research. Low statistical heterogeneity was observed across the included studies (I^2^ = 0, *P* = 0.6605; Supplementary Figure S2A). The results demonstrated that compared with CWM monotherapy, combined treatment of CWM with all included TCMIs significantly improved the clinical efficacy in patients, with statistically significant intergroup differences. Pairwise comparisons among different TCMIs revealed no statistically significant differences between any two intervention regimens (Figure 5A).

#### 3.5.4 Cardiac structure and function indicators

##### 3.5.4.1 LVEF

**Figure 6:**
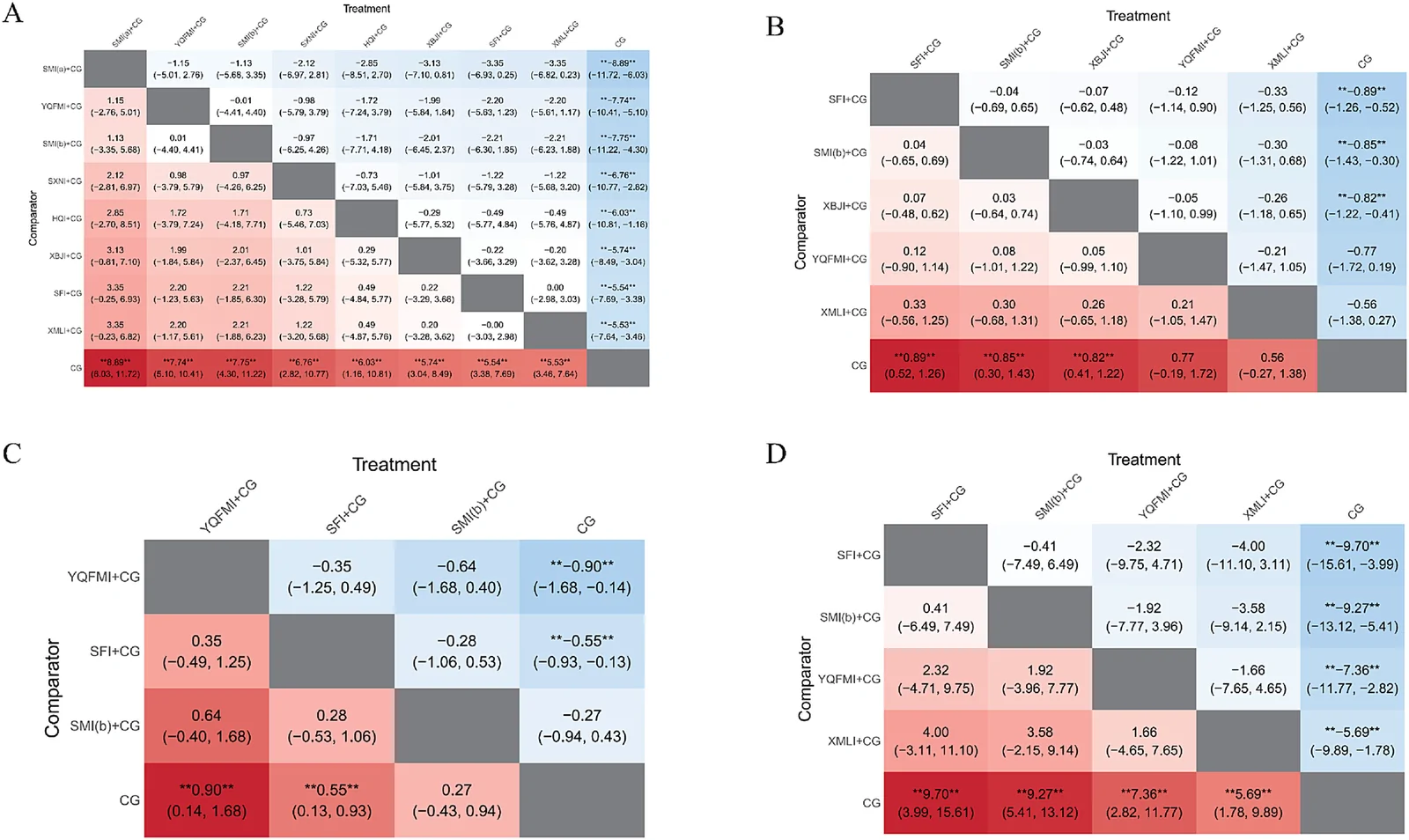
Heatmaps of cardiac structure and function indicators. **(A)** LVEF; **(B)** CO; **(C)** CI; **(D)** SV. SMI(a): Shenmai Injection; SMI(b): Shengmai Injection.** indicates a statistically significant difference between the two interventions.

A total of 47 studies (17–19, 22–25, 28–30, 36–40, 42, 43, 45–51, 53, 54, 56–61, 63–77) with a total sample size of 4,559 subjects involving 8 types of TCMIs were included in the present analysis. Substantial statistical heterogeneity was detected among the included studies (I^2^ = 88.9, *P* < 0.0001; Supplementary Figure S2B). The results indicated that compared with CWM monotherapy, the combination of CWM with all included TCMIs produced a significant improvement in patients’ LVEF, with statistically significant between-group differences. Pairwise comparisons across different TCMIs showed no statistically significant differences between any two intervention regimens (Figure 6A).

##### 3.5.4.2 LVEDD

A total of 19 studies (22, 28, 30, 33, 34, 36, 39, 43, 49, 51, 53, 54, 58, 67–69, 71, 72, 75) with a total sample size of 2,107 subjects involving 7 types of TCMIs were included in the present analysis. Substantial statistical heterogeneity was detected among the included studies (I^2^ = 67, *P* < 0.0001; Supplementary Figure S2C). The results indicated that compared with CWM monotherapy, the combination of CWM with all included TCMIs could significantly reduced patients’ LVEDD, with statistically significant between-group differences. Pairwise comparisons of different TCMIs demonstrated that SXNI, XBJI and SMI(a) combined with CWM yielded superior efficacy compared with the SFI plus CWM regimen; no statistically significant differences were observed in pairwise comparisons among the remaining intervention regimens (Figure 5B).

##### 3.5.4.3 LVESD

A total of 7 studies (34, 36, 39, 43, 49, 58, 68) with a total sample size of 704 subjects involving 5 types of TCMIs were included in this analysis. Substantial statistical heterogeneity was observed among the included studies (I^2^ = 83, *P* < 0.0001; Supplementary Figure S2D). The results indicated that compared with CWM monotherapy, SMI(a) combined with CWM and XMLI combined with CWM both significantly improved patients’ LVESD, with statistically significant between-group differences. Pairwise comparisons across different TCMIs revealed no statistically significant differences between any two intervention regimens (Figure 5C).

##### 3.5.4.4 LVESV

A total of 5 studies (29, 34, 59, 76, 77) involving 454 subjects and 3 types of TCMIs were included in this analysis. Substantial statistical heterogeneity was identified among the included studies (I^2^ = 87.5, *P* < 0.0001; Supplementary Figure S2E). The results demonstrated that no statistically significant differences were detected when comparing TCMIs combined with CWM versus CWM monotherapy, nor in pairwise comparisons among different TCMIs (Figure 5D).

##### 3.5.4.5 LVEDV

A total of 4 studies (34, 59, 76, 77) with a total sample size of 384 subjects involving 3 types of TCMIs were included in this analysis. Substantial statistical heterogeneity was observed across the included studies (I^2^ = 82.1, *P* = 0.0008; Supplementary Figure S2F). The results indicated that compared with CWM monotherapy, HQI combined with CWM significantly improved patients’ LVEDV, with statistically significant between-group differences. Pairwise comparisons of different TCMIs revealed no statistically significant differences between any two intervention regimens (Figure 5E).

##### 3.5.4.6 CO

A total of 12 studies (17–19, 27, 39, 60, 63, 64, 66, 68–70) enrolling 1,021 subjects and involving 5 types of TCMIs were included in this analysis. Substantial statistical heterogeneity was detected among the included studies (I^2^ = 95.4, *P* < 0.0001; Supplementary Figure S2G). The results showed that compared with CWM monotherapy, SFI, SMI(b) and XBJI combined with CWM significantly elevated patients’ CO, with statistically significant between-group differences. Pairwise comparisons of different TCMIs indicated no statistically significant differences between any two intervention regimens (Figure 6B).

##### 3.5.4.7 CI

A total of 5 studies (17, 19, 27, 60, 70) with a total sample size of 382 subjects involving 3 types of TCMIs were included in this analysis. Substantial statistical heterogeneity was observed across the included studies (I^2^ = 91.4, *P* < 0.0001; Supplementary Figure S2H). The results indicated that compared with CWM monotherapy, YQFMI combined with CWM significantly increased patients’ CI, with statistically significant between-group differences. Pairwise comparisons of different TCMIs revealed no statistically significant differences between any two intervention regimens (Figure 6C).

##### 3.5.4.8 SV

A total of 8 studies (27, 39, 43, 60, 61, 69, 70, 72) involving 826 subjects and 4 types of TCMIs were included in this analysis. Substantial statistical heterogeneity was detected among the included studies (I^2^ = 86, *P* < 0.0001; Supplementary Figure S2I). The results indicated that compared with CWM monotherapy, the combination of CWM with all included TCMIs significantly elevated patients’ SV, with statistically significant between-group differences. Pairwise comparisons of different TCMIs showed no statistically significant differences between any two intervention regimens (Figure 6D).

#### 3.5.5 Hemodynamic indicators

##### 3.5.5.1 HR

**Figure 7:**
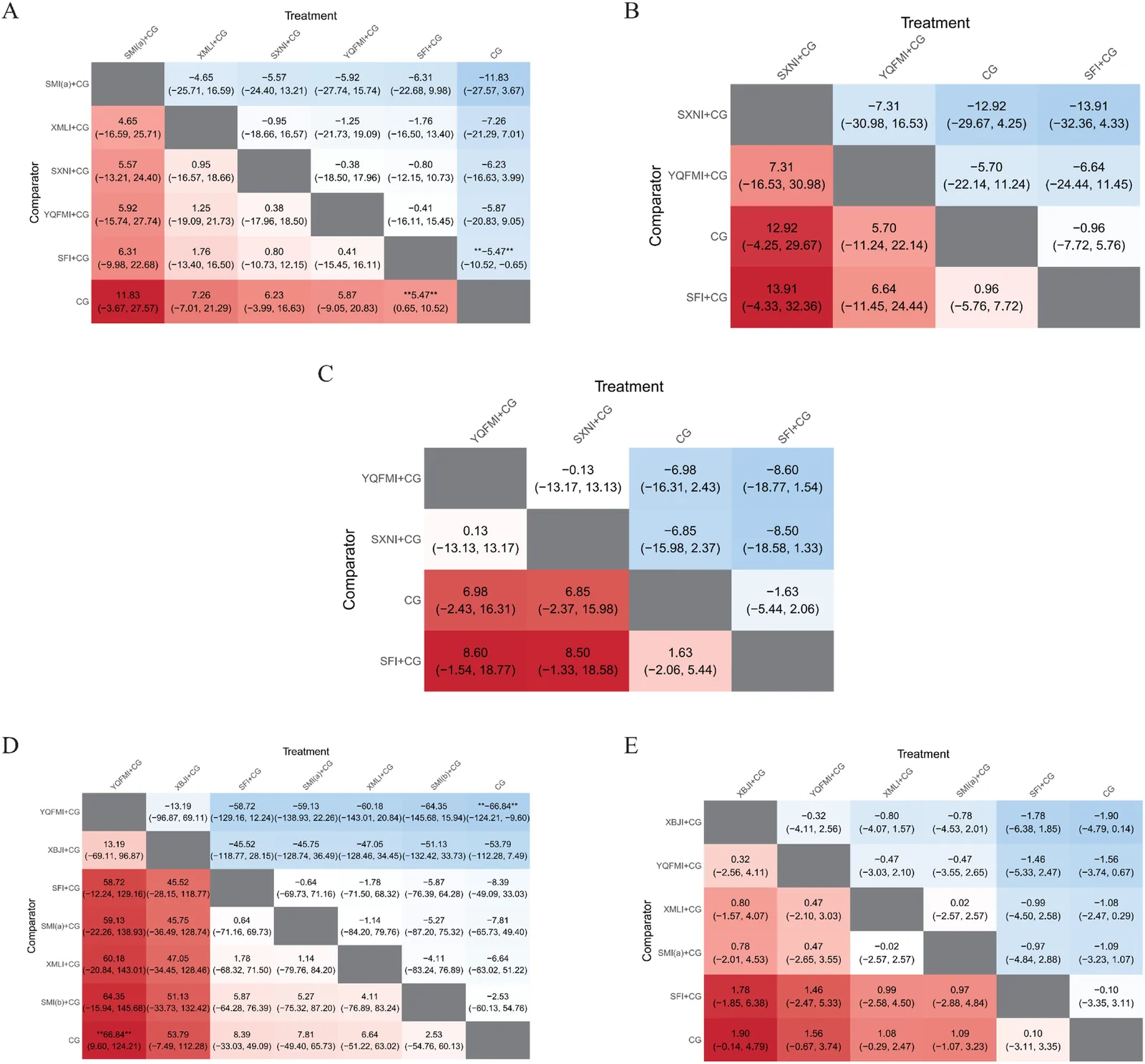
Heatmaps of hemodynamic indicators and myocardial injury biomarkers. **(A)** HR; **(B)** SBP; **(C)** DBP; **(D)** CK-MB; **(E)**cTnI. SMI(a): Shenmai Injection; SMI(b): Shengmai Injection.** indicates a statistically significant difference between the two interventions.

A total of 15 studies (17, 19–21, 25, 28–32, 39, 48, 62, 74, 75) with a total sample size of 1,287 subjects involving 5 types of TCMIs were included in this analysis. Substantial statistical heterogeneity was detected among the included studies (I^2^ = 93.1, *P* < 0.0001; Supplementary Figure S2J). The results indicated that compared with CWM monotherapy, SFI combined with CWM significantly reduced patients’ HR, with statistically significant between-group differences. Pairwise comparisons of different TCMIs revealed no statistically significant differences between any two intervention regimens (Figure 7A).

##### 3.5.5.2 SBP

A total of 8 studies (20, 21, 28, 30–32, 62, 74) involving 723 subjects and 3 types of TCMIs were included in this analysis. Substantial statistical heterogeneity was observed across the included studies (I^2^ = 90.3, *P* < 0.0001; Supplementary Figure S2K). The results demonstrated that no statistically significant differences were found when comparing TCMIs combined with CWM versus CWM monotherapy, as well as in pairwise comparisons among different TCMIs (Figure 7B).

##### 3.5.5.3 DBP

A total of 8 studies (20, 21, 28, 30–32, 62, 74) involving 723 subjects and 3 types of TCMIs were included in this analysis. Substantial statistical heterogeneity was observed across the included studies (I^2^ = 94.5, *P* < 0.0001; Supplementary Figure S2L). The results demonstrated that no statistically significant differences were detected in comparisons between TCMIs plus CWM versus CWM monotherapy, as well as in pairwise comparisons among different TCMIs (Figure 7C).

#### 3.5.6 Myocardial injury biomarkers

##### 3.5.6.1 CK-MB

A total of 7 studies (22, 34, 44, 49, 60, 67, 69) with a total sample size of 888 subjects involving 8 types of TCMIs were included in this analysis. Substantial statistical heterogeneity was detected among the included studies (I^2^ = 89.3, *P* < 0.0001; Supplementary Figure S2M). The results indicated that compared with CWM monotherapy, YQFMI combined with CWM significantly improved clinical efficacy in patients, with statistically significant between-group differences. Pairwise comparisons of different TCMIs revealed no statistically significant differences between any two intervention regimens (Figure 7D).

##### 3.5.6.2 Ctni

A total of 12 studies (34, 37–39, 42, 46, 49, 50, 57, 60, 67, 68) involving 1,246 subjects and 8 types of TCMIs were included in this analysis. Substantial statistical heterogeneity was observed across the included studies (I^2^ = 94.4, *P* < 0.0001; Supplementary Figure S2P). The results demonstrated that no statistically significant differences were identified in comparisons between TCMIs combined with CWM versus CWM monotherapy, nor in pairwise comparisons among different TCMIs (Figure 7E).

#### 3.5.7 Heart failure biomarkers

##### 3.5.7.1 BNP

**Figure 8:**
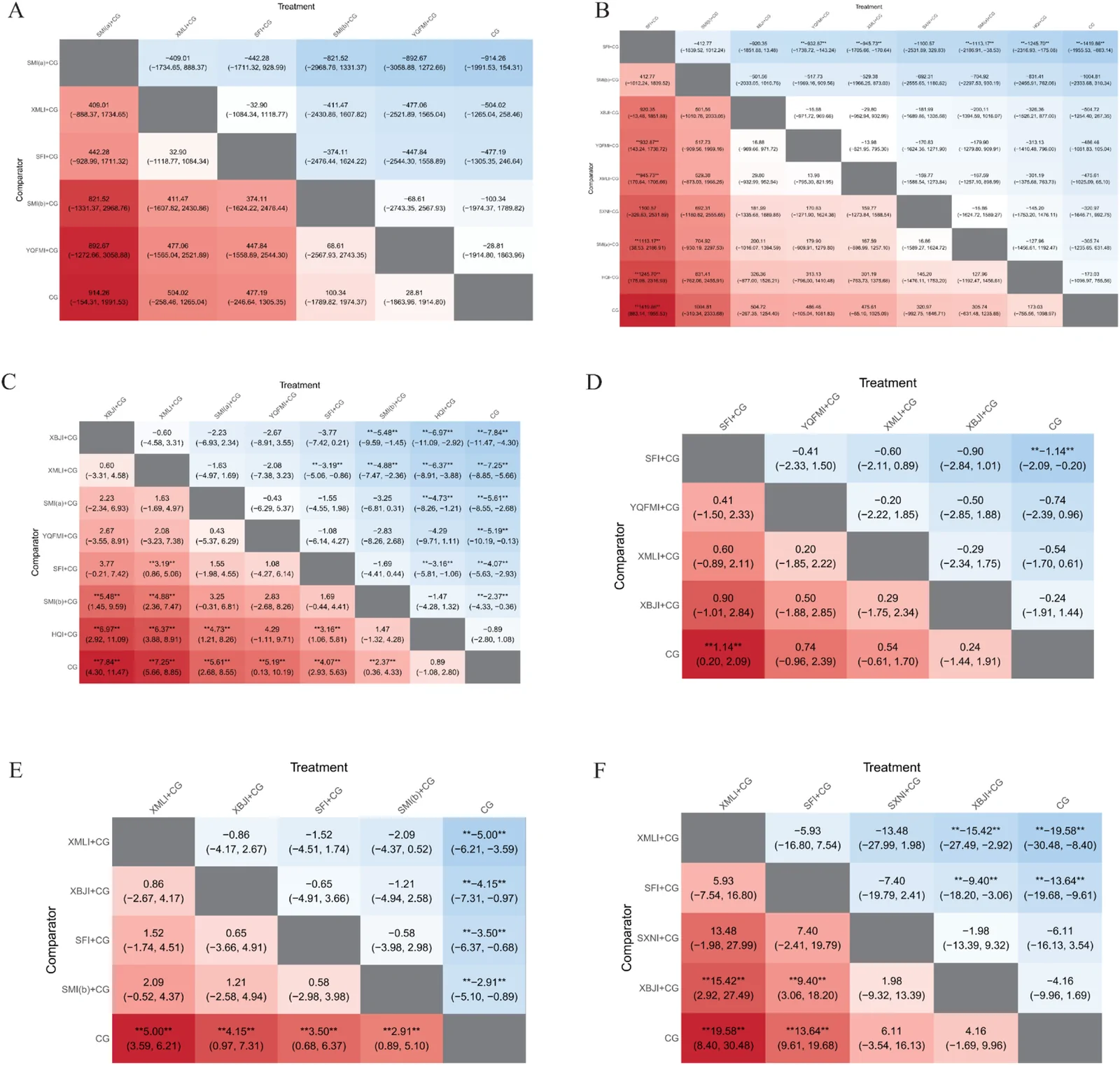
Heatmaps of heart failure biomarkers and inflammatory and endothelial function indicators. **(A)** BNP; **(B)** NT-proBNP; **(C)** hs-CRP; **(D)** TNF-α; **(E)**IL-6; **(F)** ET-1. SMI(a): Shenmai Injection; SMI(b): Shengmai Injection. ** indicates a statistically significant difference between the two interventions.

A total of 17 studies (17, 18, 20, 21, 25, 26, 35, 37, 39, 40, 42, 47, 51, 53, 54, 58, 71) with a total sample size of 1,636 subjects involving 5 types of TCMIs were included in this analysis. Substantial statistical heterogeneity was detected among the included studies (I^2^ = 97.1, *P* < 0.0001; Supplementary Figure S2N). The results demonstrated that no statistically significant differences were found when comparing TCMIs combined with CWM versus CWM monotherapy, as well as in pairwise comparisons among different TCMIs (Figure 8A).

##### 3.5.7.2 NT-proBNP

A total of 26 studies (16, 22, 28–30, 34, 36, 38, 43–46, 49, 50, 56, 57, 60–62, 65, 67–69, 75–77) enrolling 2,654 subjects and involving 8 types of TCMIs were included in this analysis. Substantial statistical heterogeneity was observed across the included studies (I2 = 96.9, *P* < 0.0001; Supplementary Figure S2O). The results indicated that compared with CWM monotherapy, SFI combined with CWM significantly decreased patients’ NT-proBNP levels, with statistically significant between-group differences. Pairwise comparisons of different TCMIs showed that the efficacy of SFI plus CWM was superior to regimens consisting of YQFMI, XMLI, SMI(a), or HQI combined with CWM; no statistically significant differences were detected in pairwise comparisons among the remaining intervention strategies (Figure 8B).

#### 3.5.8 Inflammatory and endothelial function indicators

##### 3.5.8.1 hs-CRP

A total of 18 studies (17, 19, 26, 28–30, 34, 37, 42–44, 50, 56, 64, 69, 70, 76, 77) involving 1,529 subjects and 7 types of TCMIs were included in this analysis. Substantial statistical heterogeneity was detected among the included studies (I^2^ = 89.5, *P* < 0.0001; Supplementary Figure S2Q). The results indicated that compared with CWM monotherapy, all TCMIs combined with CWM significantly reduced patients’ hs-CRP levels except HQI, with statistically significant between-group differences. Pairwise comparisons of different TCMIs revealed the following findings: the efficacy of XBJI plus CWM was superior to that of SMI(b) plus CWM; XMLI combined with CWM was more effective than SFI plus CWM and SMI(b) plus CWM regimens. In addition, XBJI, XMLI, SMI(a), and SFI combined with CWM each exhibited better therapeutic effects than HQI plus CWM. No statistically significant differences were observed in pairwise comparisons among the remaining intervention regimens (Figure 8C).

##### 3.5.8.2 TNF-α

A total of 7 studies (17, 19, 34, 40, 43, 60, 64) with a total sample size of 574 subjects involving 4 types of TCMIs were included in this analysis. Substantial statistical heterogeneity was observed across the included studies (I^2^ = 92.6, *P* < 0.0001; Supplementary Figure S2R). The results indicated that compared with CWM monotherapy, SFI combined with CWM significantly decreased patients’ TNF-α levels, with statistically significant between-group differences. Pairwise comparisons of different TCMIs revealed no statistically significant differences between any two intervention regimens (Figure 8D).

##### 3.5.8.3 IL-6

A total of 10 studies (34–36, 40, 42–44, 64, 69, 70) involving 914 subjects and 4 types of TCMIs were included in this analysis. Substantial statistical heterogeneity was detected among the included studies (I^2^ = 82.3, *P* < 0.0001; Supplementary Figure S2). The results indicated that compared with CWM monotherapy, all TCMIs combined with CWM significantly reduced patients’ IL-6 levels, with statistically significant between-group differences. Pairwise comparisons of different TCMIs revealed no statistically significant differences between any two intervention regimens (Figure 8E).

##### 3.5.8.4 ET-1

A total of 10 studies (16, 22, 28–30, 37, 63, 64, 66, 75) with a total sample size of 1,043 subjects involving 4 types of TCMIs were included in this analysis. Moderate statistical heterogeneity was detected among the included studies (I^2^ = 56.9, *P* = 0.0131; Supplementary Figure S2T). The results indicated that compared with CWM monotherapy, XMLI plus CWM and SFI plus CWM significantly reduced patients’ ET-1 levels, with statistically significant between-group differences. Pairwise comparisons of different TCMIs demonstrated that the therapeutic efficacy of XMLI combined with CWM and SFI combined with CWM was superior to that of XBJI combined with CWM; no statistically significant differences were found in pairwise comparisons among the remaining intervention regimens (Figure 8F).

#### 3.5.9 Incidence of MACE

A total of 11 studies (22, 24, 25, 28–30, 49, 52–54, 61) involving 1,233 subjects and 3 types of TCMIs were included in this analysis. Low statistical heterogeneity was observed across the included studies (I^2^ = 0, *P* = 0.6071; Supplementary Figure S2U). The results indicated that compared with CWM monotherapy, all TCMIs combined with CWM significantly reduced the incidence of MACE, with statistically significant between-group differences. Pairwise comparisons of different TCMIs revealed no statistically significant differences between any two intervention regimens (Figure 5F).

#### 3.5.10 Adverse reactions

Aggregate analysis of adverse reaction data from the included studies showed that adverse events reported in both groups mainly involved the circulatory system, digestive system, skin and adnexa, hepatorenal function, and nervous system (Table 2). Among them, hypotension was the most common circulatory adverse reaction, followed by arrhythmia (including ventricular arrhythmia), tachycardia, palpitations and chest tightness. Digestive system adverse reactions were relatively prevalent, mainly manifested as nausea and vomiting, abdominal pain, diarrhea, abdominal distension, constipation and various gastrointestinal discomforts. Skin-related adverse reactions included rash, skin pruritus and angioedema. Abnormalities related to liver and kidney function were observed in multiple studies, such as abnormal liver and kidney function indices, impaired liver and kidney function, liver function injury and renal function impairment. Nervous system adverse reactions were predominantly dizziness and headache. Overall, adverse reactions in both groups were mild and mostly self-limiting. No concentrated occurrence of severe adverse events was found in the studies, indicating that the intervention regimen possessed favorable overall safety.

**Table 2:** Incidence of adverse reactions.

First author and year	Adverse reactions		
	Experimental group	Control group	
ZHANG YH et al., 2019 (26)	2 cases of tachycardia; heart rate normalized after *β*-blocker adjustment		None
ZHAO J et al., 2016 (28)	1 case of hypotension; 2 cases of ventricular arrhythmia		8 cases of hypotension; 8 cases of ventricular arrhythmia
WANG HY et al., 2018 (30)	1 case of hypotension; 2 cases of ventricular arrhythmia		8 cases of hypotension; 8 cases of ventricular arrhythmia
LIU DS et al., 2020 (38)	2 cases of skin pruritus; 1 case of gastrointestinal reaction		1 case of skin pruritus
WANG WG et al., 2024 (44)	4 cases of nausea and vomiting; 2 cases of dizziness and headache; 1 case of abdominal distension and diarrhea		2 cases of nausea and vomiting; 1 case of dizziness and headache; 1 case of abdominal distension and diarrhea
LYU YJ et al., 2021 (50)	4 cases of abnormal liver and kidney function		3 cases of abnormal liver and kidney function
LI XY, 2019 (55)	None		None
LI X et al., 2021 (58)	1 case of constipation		2 cases of abdominal pain
GONG KW et al., 2023 (60)	2 cases of arrhythmia; 2 cases of hypotension; 1 case of impaired liver and kidney function		2 cases of arrhythmia; 1 case of hypotension
LI XF et al., 2016 (61)	None		None
LU X et al., 2024 (65)	1 case of angioedema; 1 case of rash; 1 case of hypotension; 1 case of liver function impairment		2 cases of angioedema; 2 cases of hypotension; 2 cases of liver function impairment
WANG QF, 2024 (66)	1 case of hypotension; 1 case of rash; 1 case of angioedema		4 cases of hypotension; 3 cases of rash; 2 cases of angioedema; 1 case of renal function impairment
ZHANG J et al., 2025b (67)	1 case of headache; 5 cases of gastrointestinal discomfort		2 cases of headache; 2 cases of hypotension; 1 case of gastrointestinal discomfort
LI MJ, 2025 (68)	1 case of digestive system symptoms; 1 case of dizziness; 1 case of rash		1 case of impaired liver and kidney function; 1 case of digestive system symptoms; 2 cases of dizziness; 1 case of rash
LU DX et al., 2022 (70)	2 cases of nausea; 2 cases of rash		2 cases of nausea; 2 cases of headache; 1 case of palpitations
XIAN W, 2019 (76)	2 cases of vomiting; 3 cases of chest tightness; 4 cases of hypotension		3 cases of vomiting; 2 cases of chest tightness; 2 cases of hypotension

#### 3.5.11 Ranking based on SUCRA values

**Figure 9:**
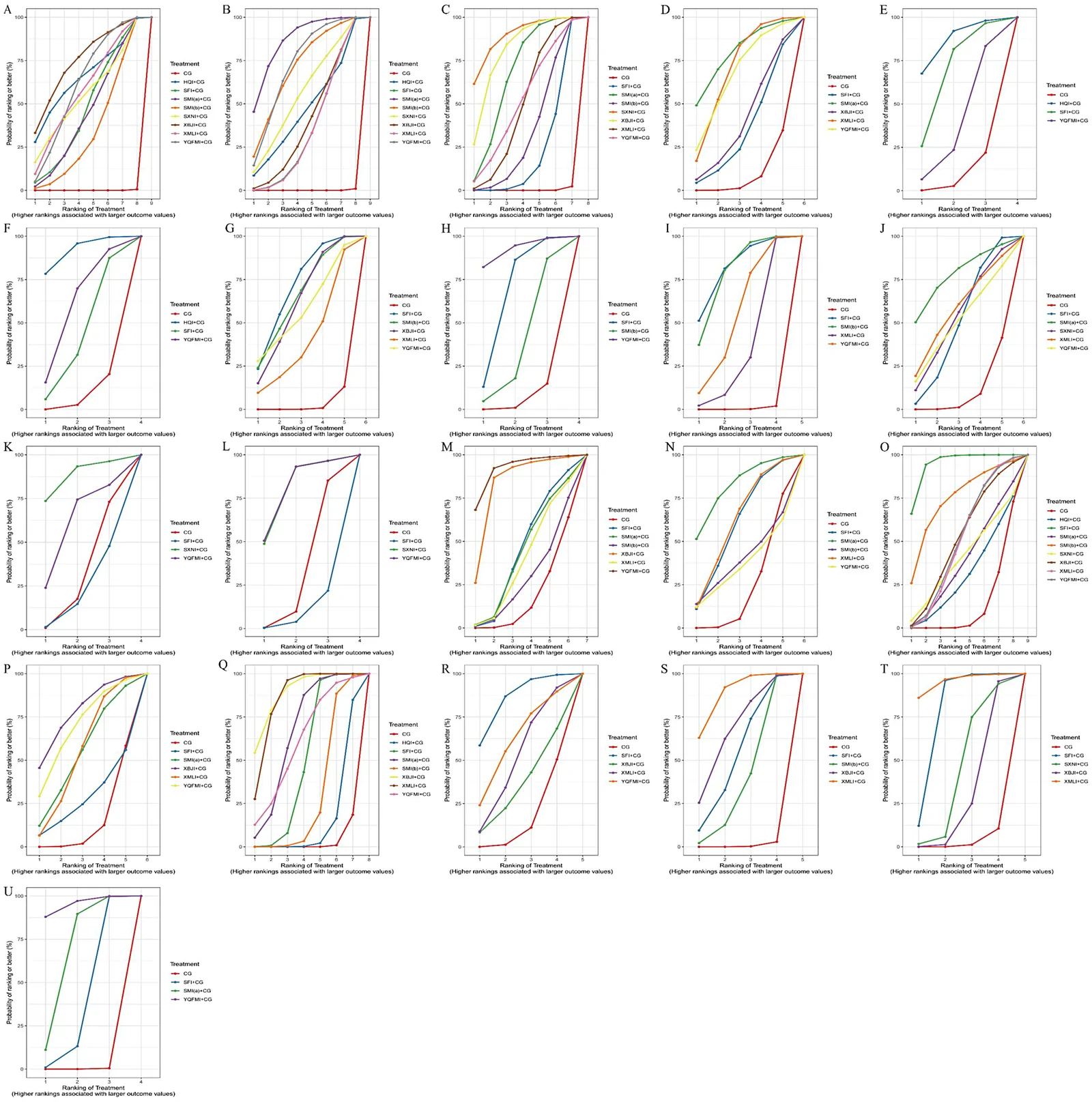
Cumulative probability ranking of TCMIs + CG for the treatment of post-AMI HF. **(A)** Clinical efficacy; **(B)** LVEF; **(C)** LVEDD; **(D)** LVESD; **(E)** LVESV; **(F)** LVEDV; **(G)** CO; **(H)** CI; **(I)** SV; **(J)** HR; **(K)** SBP; **(L)** DBP; **(M)** CK-MB; **(N)** BNP; **(O)** NT-proBNP; **(P)** cTnI; **(Q)** hs-CRP; **(R)** TNF-α; **(S)** IL-6; **(T)** ET-1; **(U)** MACE. SMI(a): Shenmai Injection; SMI(b): Shengmai Injection.

The results of the SUCRA indicated that SFI plus CWM achieved the optimal efficacy in elevating CO and SV, as well as reducing NT-proBNP and TNF-α. XMLI ranked favorably in lowering IL-6 and ET-1. SMI obtained the highest SUCRA rankings in boosting LVEF, ameliorating LVESD, HR and BNP. YQFMI presented superior rankings in improving CI, DBP, CK-MB and reducing the occurrence of MACE. XBJI occupied the top SUCRA position in improving overall clinical efficacy and decreasing cTnI and hs-CRP. SXNI ranked first in adjusting LVEDD and SBP, while HQI yielded the best outcomes in improving LVESD and LVEDV (Figure 9, Supplementary Table S2).

#### 3.5.12 Assessment of publication bias

**Figure 10:**
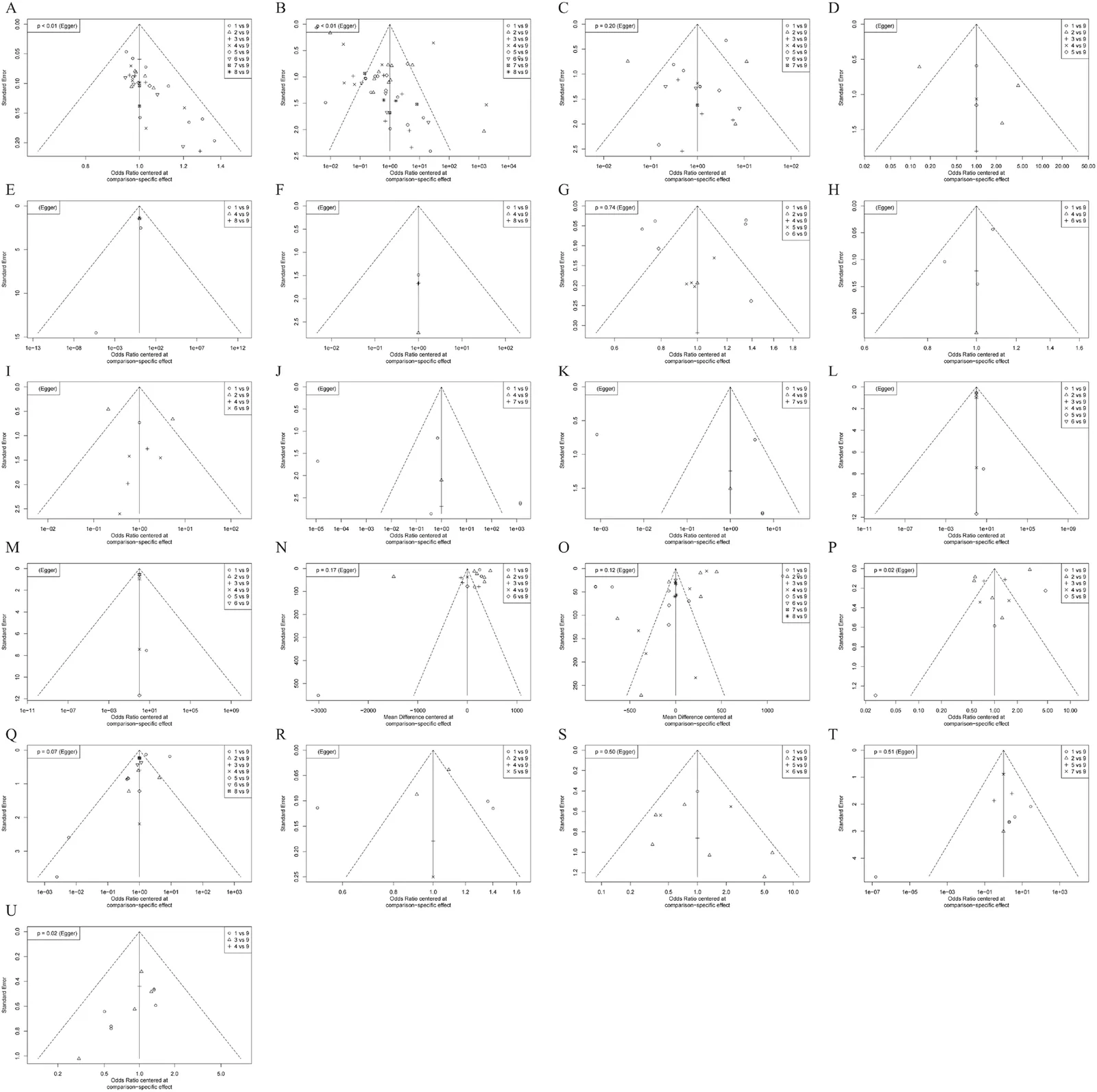
Calibrated funnel plots of various outcome indicators for TCMIs + CG in the treatment of post-AMI HF. 1.SFI + CG; 2.XMLI + CG; 3.SMI(a)+CG; 4.YQFMI + CG; 5.XBJI + CG; 6.SMI(b)+CG; 7. SXNI + CG; 8 HQI + CG; 9.CG. **(A)** Clinical efficacy; **(B)** LVEF; **(C)** LVEDD; **(D)** LVESD; **(E)** LVESV; **(F)** LVEDV; **(G)** CO; **(H)** CI; **(I)** SV; **(J)** HR; **(K)** SBP; **(L)** DBP; **(M)** CK-MB; **(N)** BNP; **(O)** NT-proBNP; **(P)** cTnI; **(Q)** hs-CRP; **(R)** TNF-α; **(S)** IL-6; **(T)** ET-1; **(U)** MACE.

To evaluate potential publication bias and small-study effects, comparison-adjusted funnel plots were generated for 21 outcome indicators, together with Egger's regression tests for quantitative assessment (Figure 10). The results showed that the distribution patterns of funnel plots varied across different outcomes. Several outcomes (e.g., Clinical efficacy and LVEF) exhibited evident asymmetry, with scattered study points deviating from the central line, and Egger's tests indicated statistically significant results (*P* < 0.05 or *P* < 0.01), suggesting the presence of potential small-study effects or publication bias. In contrast, some outcomes (e.g., CO, CI, TNF-α and IL-6) demonstrated relatively symmetrical funnel plot distributions, with study points evenly distributed around the central line and no obvious systematic deviation, indicating a lower risk of publication bias in these outcomes.

Overall, the results suggest that publication bias varies across different outcomes in this network meta-analysis. While not all endpoints were affected, certain outcomes may still be influenced by small-study effects or selective publication. Therefore, the pooled results should be interpreted with caution considering the potential impact of publication bias.

#### 3.5.13 Meta-regression analysis

**Figure 11:**
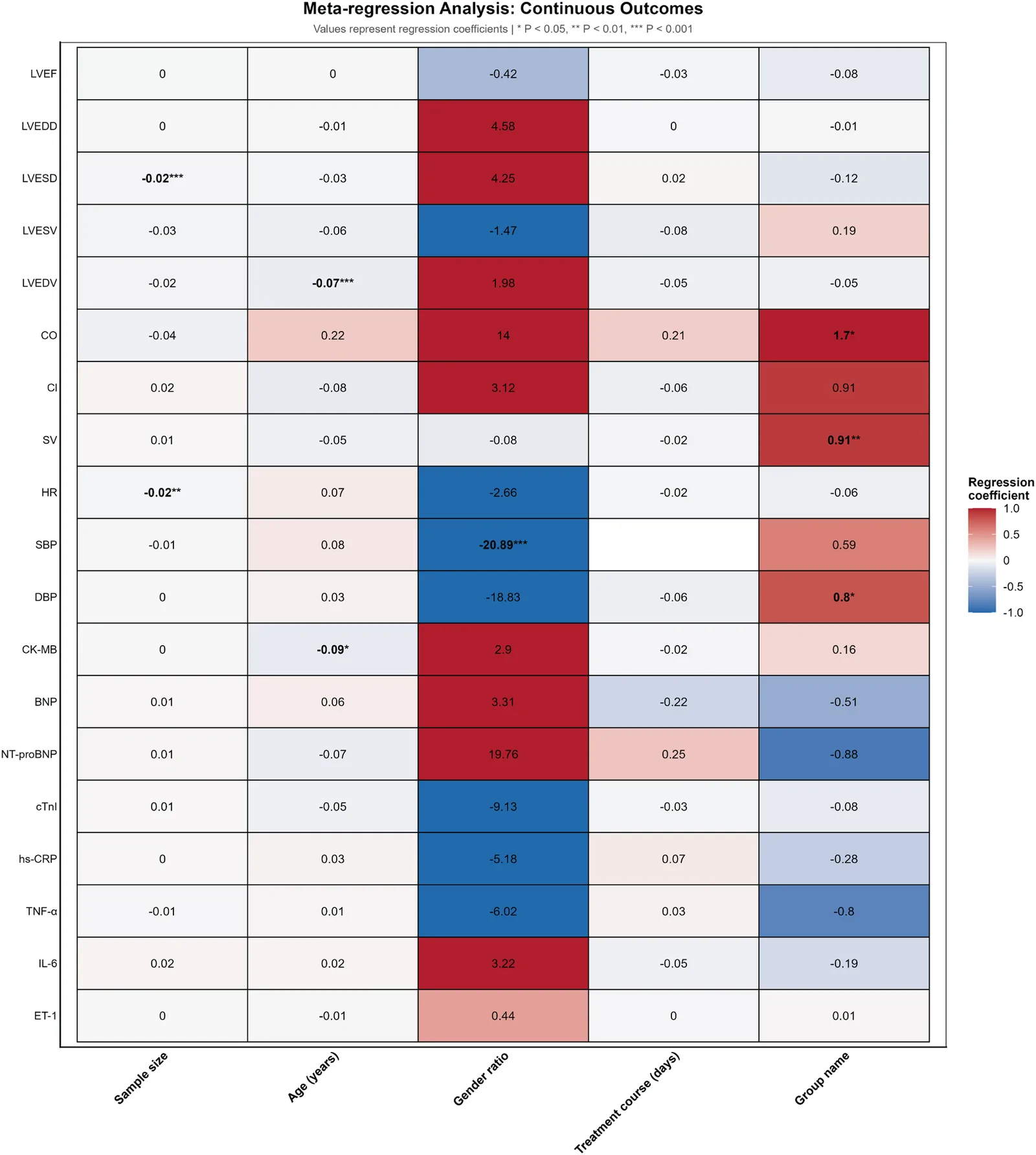
Meta-regression analyses of age, sample size, gender ratio, treatment course and intervention type.

Meta-regression analyses were performed to explore potential sources of heterogeneity across continuous outcomes in this network meta-analysis. The covariates included sample size, mean age, gender ratio, treatment duration, and intervention classification (group name). The results (Figure 11) indicated that intervention classification (group name), representing different categories of traditional Chinese medicine injections based on pharmacological efficacy, was the most influential moderator across multiple outcomes.

Specifically, significant effects were observed in cardiac structural parameters, including LVEDD (*β* = 4.58), LVESD (*β* = 4.25), LVEDV (*β* = 1.98), and LVESV (*β* = −1.47), suggesting substantial between-group differences in ventricular remodeling outcomes. For cardiac function indicators, group classification showed pronounced effects on CO (*β* = 14.00), CI (*β* = 3.12), and SV (*β* = −0.08), indicating notable heterogeneity across intervention categories. In terms of hemodynamic variables, SBP (*β* = −20.89) and DBP (*β* = −18.83) were strongly influenced by intervention classification, reflecting variability in blood pressure-related outcomes. For myocardial injury and inflammatory markers, CK-MB (*β* = 2.9), IL-6 (*β* = 3.22), and ET-1 (*β* = 0.44) showed positive associations with group classification, whereas TNF-α (*β* = −6.02) and hs-CRP (*β* = −5.18) showed negative associations. Among covariates, age demonstrated moderate effects on LVESD (*β* = −0.03) and LVEDV (*β* = −0.07), while gender ratio showed limited but observable influence on CO (*β* = 1.7*) and SV (*β* = 0.91**). Sample size and treatment duration exhibited relatively weak and inconsistent effects across outcomes. Overall, intervention classification (group name) was identified as the primary source of heterogeneity in this analysis.

#### 3.5.14 Consistency, sensitivity analysis and robustness

Consistency between direct and indirect evidence was assessed using node-splitting analysis. The results showed no significant inconsistency in most comparisons (*P* > 0.05), indicating good agreement between direct and indirect evidence, and supporting the use of a consistency model. Sensitivity analyses were performed by sequentially excluding studies with extreme effect sizes and high risk of bias. The pooled results remained stable across all major outcomes, indicating that the findings were robust and not substantially influenced by individual studies. Overall consistency across multiple outcome domains, including cardiac function, hemodynamic parameters, myocardial injury markers, and inflammatory factors, further supported the reliability of the network estimates. Taken together, although moderate heterogeneity was observed across studies, the overall network structure demonstrated acceptable stability and robustness.

#### 3.5.15 CINeMA assessment of evidence certainty

The CINeMA framework (78–80) was applied to evaluate the certainty of evidence for the primary outcomes across six domains, including within-study bias, reporting bias, indirectness, imprecision, heterogeneity, and inconsistency. The results indicated that the overall quality of evidence ranged from low to very low across most outcomes (Supplementary Table S3). The main reasons for downgrading the certainty of evidence were as follows.

First, all included studies were single-center randomized controlled trials conducted in China, and most were not prospectively registered. Methodological reporting was generally insufficient, with inadequate descriptions of random sequence generation, allocation concealment, and blinding procedures, indicating a relatively high risk of within-study bias.

Second, the evidence network was a star-shaped structure, with no direct head-to-head comparisons among different interventions. Therefore, the analysis relied predominantly on indirect evidence, increasing uncertainty and limiting causal inference.

Third, meta-regression results suggested that intervention classification (group name) was the primary source of heterogeneity. Notably, different categories of traditional Chinese medicine injections are theoretically associated with distinct traditional Chinese medicine syndrome (pattern) types. However, syndrome differentiation information was insufficiently reported and not adequately stratified in the included studies, which may have introduced additional unmeasured clinical heterogeneity and increased indirectness.

In addition, substantial clinical heterogeneity was observed across outcomes, and some effect estimates were associated with wide confidence intervals, further reducing statistical precision. Although sensitivity analyses suggested that the overall results were relatively stable and no significant inconsistency was detected, the evidence was still primarily based on indirect comparisons, indicating an exploratory nature of the findings.

In conclusion, the overall certainty of evidence was low to very low. Therefore, the findings should be interpreted as exploratory rather than confirmatory. Future studies should focus on multicenter, prospective, well-registered randomized controlled trials, with adequate syndrome stratification and direct head-to-head comparisons between interventions to improve the certainty and clinical applicability of the evidence.

## 4 Discussion

This study employed a Bayesian NMA to systematically compare the efficacy and safety of eight TCMIs combined with CWM in patients with post-AMI HF. A total of 62 RCTs involving 5,922 participants were included. Outcomes were comprehensively evaluated across multiple dimensions, including cardiac structure and function, hemodynamics, myocardial injury biomarkers, heart failure biomarkers, inflammatory and endothelial indicators, MACE, and adverse reactions. Based on SUCRA cumulative probability ranking, the relative advantages of each injection across different outcomes were determined, providing preliminary evidence for individualized integrated traditional Chinese and Western medicine management of post-AMI heart failure.

### 4.1 Pathophysiology of post-AMI HF and theoretical basis of integrated TCM and Western medicine therapy

AMI can lead to myocardial ischemic necrosis, subsequently triggering a cascade of pathological processes, including marked activation of inflammatory responses, persistent overactivation of the neuroendocrine system, progressive ventricular remodeling, and coronary microcirculatory dysfunction. These processes ultimately result in sustained deterioration of cardiac systolic and diastolic function and significantly increase the risk of rehospitalization and cardiovascular mortality (81–84). Although standard CWM, including antiplatelet agents, statins, renin-angiotensin-aldosterone system (RAAS) inhibitors, beta-blockers, and diuretics, can partially reverse ventricular remodeling and improve short-term prognosis, a considerable proportion of patients still experience persistent cardiac dysfunction and recurrent heart failure symptoms. This suggests that a single therapeutic strategy is insufficient to fully interrupt the pathological progression driven by sustained inflammatory activation and impaired myocardial energy metabolism (81, 85, 86).

Within the framework of TCM, post-AMI HF is categorized as “chest bi syndrome,” “palpitation,” and “edema.” The core pathogenesis is characterized by deficiency of heart qi and heart yang as the root, with blood stasis and fluid retention as the manifestations. In prolonged disease progression, damp-heat and toxin accumulation may further develop. Chinese medicine injections, characterized by intravenous administration, rapid onset, and multi-target regulatory effects, can simultaneously exert qi-tonifying, yang-warming, blood-activating, diuresis-promoting, and detoxifying actions. When combined with standard Western medical therapy, they may produce synergistic effects across key pathological processes, including inflammation regulation, myocardial energy metabolism, and ventricular remodeling.

Previous systematic review (15) have also suggested that combined therapy with TCMIs may be more effective than Western medicine alone in improving cardiac function, reducing inflammatory levels, and decreasing the incidence of MACE. The findings of this study further support the adjunctive therapeutic value of Chinese medicine injections in the comprehensive management of post-AMI HF.

### 4.2 Stratified efficacy and mechanism correspondence of different TCMIs

The SUCRA ranking results indicated that different TCMIs exhibited heterogeneous advantages across various outcome indicators. Their performance showed a certain degree of consistency with TCM therapeutic principles and modern pharmacological mechanisms, and also demonstrated differences in clinical applicability among patient subgroups.

#### 4.2.1 SFI

SFI demonstrated relatively prominent effects in improving CO, SV, and reducing NT-proBNP and TNF-α levels. Its core TCM function is to warm and tonify original yang and reinforce qi to strengthen the heart. Modern pharmacological studies suggest that it can enhance myocardial contractility, improve myocardial energy metabolism, and inhibit the NF-*κ*B inflammatory signaling pathway, thereby alleviating cardiac dysfunction induced by excessive neuroendocrine activation (87, 88). It may therefore be more suitable for post-AMI HF patients with markedly reduced cardiac function and significant inflammatory activation.

#### 4.2.2 XMLI

XMLI showed more pronounced effects in improving IL-6 and ET-1 levels. Its mechanism of action is mainly associated with protection of vascular endothelial function and inhibition of inflammatory responses. It may reduce ET-1-mediated vasoconstriction, improve coronary microcirculation, and interrupt the pathological cascade of inflammation–endothelial injury–ventricular remodeling. It is therefore more suitable for patients with prominent inflammatory responses and endothelial dysfunction.

#### 4.2.3 SMI(a)

SMI(a) demonstrated superior effects in improving LVEF, reducing left ventricular internal diameter, and slowing HR. Its formulation is primarily composed of ginseng and ophiopogon, with the function of tonifying qi and nourishing yin. Pharmacologically, it may stabilize cardiomyocyte membrane function, reduce myocardial oxygen consumption, and improve diastolic dysfunction (89, 90). It shows relatively stable efficacy in HF with reduced ejection fraction.

#### 4.2.4 YQFMI

YQFMI showed favorable effects in improving CI, regulating DBP, reducing CK-MB, and decreasing MACE. It may improve hemodynamic stability and reduce myocardial enzyme release, thereby lowering early cardiovascular risk. It may be more suitable for patients in the early stage of myocardial infarction with hemodynamic instability.

#### 4.2.5 XBJI

XBJI showed relatively high SUCRA values in reducing cTnI and hs-CRP. Its main mechanism is considered to involve blood-activating, detoxifying, and anti-inflammatory effects. It may inhibit inflammatory cascades and reduce secondary myocardial injury following infarction. It may therefore be suitable for patients with pronounced inflammatory responses and blood stasis–toxin accumulation patterns.

#### 4.2.6 SXNI

SXNI injection demonstrated relatively better effects in improving LVEDD and reducing SBP. Its main mechanism may involve coronary vasodilation, improved myocardial perfusion, and reduced cardiac preload and afterload, thereby contributing to ventricular remodeling improvement.

#### 4.2.7 HQI

HQI showed favorable effects in improving left ventricular systolic and diastolic volumes. Its mechanism is mainly associated with tonifying qi, raising yang, and improving myocardial energy metabolism, thereby delaying ventricular dilation. It may be more suitable for patients with chronic heart failure and a longer disease course.

### 4.3 Safety evaluation of combined therapy

The pooled results from 62 included studies on adverse events indicated that, after the combination of Chinese medicine injections with standard CWM, the overall incidence of adverse reactions was relatively low. Most adverse events were mild in severity and self-limiting, and no obvious clustering of serious adverse events was observed.

The reported adverse reactions mainly involved the cardiovascular system (such as hypotension and arrhythmia), the gastrointestinal system (such as nausea and abdominal pain), and skin hypersensitivity reactions (such as rash and pruritus). A small number of cases showed transient abnormalities in liver or renal function indicators. These adverse reactions generally resolved after adjustment of infusion rate or symptomatic treatment.

The safety profile observed in this study is broadly consistent with previous findings in cardiovascular research involving Chinese medicine injections. Meanwhile, no increasing trend was observed in the incidence of MACE, including malignant arrhythmia and reinfarction, in the combination therapy group. This suggests that the combined use of Chinese medicine injections demonstrates an overall acceptable safety profile within the current evidence base. However, in clinical practice, standardized administration procedures are still required, including strict allergy screening and careful control of intravenous infusion speed, to reduce potential hypersensitivity risks.

### 4.4 Clinical implications and stratified treatment recommendations

Based on the current network meta-analysis results, TCM theory of syndrome differentiation, and existing pharmacological evidence, this study proposes stratified treatment suggestions under an exploratory evidence framework, aiming to provide preliminary guidance for future individualized clinical decision-making. However, it should be emphasized that due to the generally low quality of evidence and the predominance of indirect comparisons in the network structure, these recommendations are exploratory in nature and should not be interpreted as definitive clinical guidelines.

#### 4.4.1 General preferred strategy

For patients with post-AMI HF who present with coexisting impaired cardiac function, inflammatory activation, and ventricular remodeling, the combined use of SFI, SMI(a), and XMLI may be considered in addition to standard CWM. These three interventions demonstrated relatively balanced performance in improving cardiac function and modulating inflammation in this study. However, these findings are still based on indirect comparisons and should be interpreted with caution.

#### 4.4.2 Stratified selection based on major clinical manifestations

(1) For patients with severe cardiac dysfunction, markedly elevated NT-proBNP levels, and symptoms of cold intolerance or yang deficiency, SFI may have potential clinical value; (2) For patients with significantly elevated inflammatory markers (hs-CRP, IL-6) accompanied by blood stasis and endothelial injury, XMLI injection or Xuebijing injection may be more appropriate; (3) For patients with reduced LVEF, increased heart rate, and evident qi-yin deficiency, SMI(a) injection may provide potential benefit; (4) For patients in the early stage of myocardial infarction with elevated myocardial enzyme levels and hemodynamic instability, YQFMI may have supportive effects; (5) For patients with left ventricular enlargement and elevated systolic blood pressure, SXNI may contribute to structural improvement; (6) For patients with a longer disease course, mild to moderate chronic heart failure, and increased ventricular volume, HQI may have potential therapeutic value.

#### 4.4.3 Combined and sequential treatment strategies

For complex patients presenting with multiple pathological features simultaneously, sequential use of different TCMIs with complementary mechanisms may be considered under the guidance of a TCM practitioner. However, co-infusion of different injections should be avoided to reduce potential risks. Meanwhile, TCMIs should always be used as adjunctive therapy to standard CWM and should not replace guideline-directed pharmacological treatment strategies.

### 4.5 Limitations

Several limitations of this study should be acknowledged. First, the primary limitation of this study is that the evidence network was exclusively based on indirect comparisons. The network structure was predominantly star-shaped, with conventional Western medicine serving as the common comparator, and no direct head-to-head randomized controlled trials comparing different TCMIs were identified. Although network meta-analysis allows indirect estimation of relative treatment effects among multiple interventions, the absence of direct comparisons increases uncertainty in the comparative rankings. Therefore, the SUCRA-based hierarchy should be interpreted as exploratory rather than definitive evidence of superiority. Future well-designed head-to-head randomized controlled trials directly comparing different TCMIs are warranted to validate these findings.

Second, heterogeneity existed in the background conventional Western medical therapies among the included studies. In this study, conventional Western medicine was defined as guideline-directed medical therapy combined with additional active agents when clinically indicated, as reported in the original trials. Although the same background regimen was generally applied to both the experimental and control groups within each individual study, variations in concomitant therapies across different studies, such as recombinant human brain natriuretic peptide, dobutamine, levosimendan, dopamine, and enoxaparin, may have introduced residual clinical heterogeneity. Therefore, the potential influence of background treatment differences on indirect comparisons cannot be completely excluded.

Third, the transitivity assumption of network meta-analysis should be interpreted cautiously. Although we summarized available potential effect modifiers, including age, sex, Killip classification, PCI rate, baseline cardiac function indicators, and outcome assessment timing, several clinically important factors were incompletely reported in the original randomized controlled trials. In particular, detailed information regarding contemporary heart failure therapies, including angiotensin receptor–neprilysin inhibitors, beta-blockers, mineralocorticoid receptor antagonists, sodium-glucose cotransporter 2 inhibitors, and inotropic agents, was frequently unavailable. Therefore, residual uncertainty regarding baseline comparability among comparisons may remain.

Fourth, methodological limitations of the included randomized controlled trials may have affected the certainty of evidence. Although risk of bias was reassessed using the Cochrane Risk of Bias 2 tool in the revised analysis, many studies still had some concerns mainly due to insufficient reporting of randomization procedures, allocation concealment, blinding, and selective reporting. These limitations may reduce confidence in the estimated treatment effects and highlight the need for more rigorously designed clinical trials.

Fifth, differences in outcome definitions and measurement approaches may have contributed to additional uncertainty. For major adverse cardiovascular events (MACE), the included studies did not consistently apply identical definitions, component compositions, or follow-up durations, and standardized clinical adjudication was generally unavailable. Similarly, biomarker outcomes, including BNP/NT-proBNP, CK-MB, and cTnI, may have been affected by differences in laboratory assays, measurement procedures, and reporting methods among studies, despite efforts to extract and synthesize data according to the reported information.

Sixth, some limitations were related to the characteristics of the available evidence base. Most included trials were conducted in China, and the sample sizes of individual studies were relatively limited. In addition, several studies lacked detailed reporting of long-term clinical outcomes, and follow-up periods were often relatively short. Therefore, the generalizability of the findings to other healthcare settings and the long-term clinical benefits of TCMIs require further investigation.

Seventh, although extensive literature searching and screening procedures were performed, the possibility of publication bias cannot be completely excluded. Positive findings may be more likely to be published, and the limited number of studies available for some comparisons may have reduced the ability to detect small-study effects.

Finally, publication bias cannot be ignored. Funnel plots and Egger's test suggested potential asymmetry in some outcomes, indicating possible small-study effects and publication bias favoring positive results. Moreover, all included studies were conducted in a single country (China), which may also limit the external generalizability of the findings.

## 5 Conclusion

This Bayesian NMA systematically evaluated the efficacy and safety of eight TCMIs combined with CWM in post-AMI HF. The results indicated that all types of TCMIs may have potential benefits in improving cardiac function, inhibiting ventricular remodeling, reducing inflammatory responses, and decreasing myocardial injury biomarkers. However, there were obvious differences in efficacy among different interventions.

However, it should be emphasized that the overall quality of evidence in this study was low to very low, and the network structure was a typical star-shaped network. All effect estimates were primarily based on indirect comparisons, with no direct head-to-head evidence available. Therefore, both the pooled ranking results and comparative effect estimates are associated with a certain degree of uncertainty, and their robustness and causal inference capability are limited.

In addition, the included studies generally had limitations such as insufficient methodological quality, substantial clinical heterogeneity, and lack of TCM syndrome differentiation information. Therefore, the current evidence should be interpreted as exploratory rather than being used for definitive clinical decision-making.

In conclusion, TCMIs combined with CWM may have potential adjunctive therapeutic value in post-AMI HF; however, the current evidence is insufficient to draw definitive conclusions regarding superiority among different interventions. Further high-quality randomized controlled trials with multicenter design, larger sample sizes, strict registration, and direct head-to-head comparisons are still required to validate these findings.

## Data Availability

The datasets used in this study are derived from published literature and are available from the corresponding original publications. Requests to access these datasets should be directed to mazhanze97@126.com.
